# Assessment of Treatment Effects and Long-term Benefits in Immune Checkpoint Inhibitor Trials Using the Flexible Parametric Cure Model: A Systematic Review

**DOI:** 10.1001/jamanetworkopen.2021.39573

**Published:** 2021-12-21

**Authors:** Thomas Filleron, Marine Bachelier, Julien Mazieres, Maurice Pérol, Nicolas Meyer, Elodie Martin, Fanny Mathevet, Jean-Yves Dauxois, Raphael Porcher, Jean-Pierre Delord

**Affiliations:** 1 Department of Biostatistics, Institut Claudius Regaud, Institut Universitaire du Cancer Toulouse, Toulouse, France; 2 Department of Pneumology, Centre Hospitalier Universitaire de Toulouse Larrey, Toulouse, France; 3 Department of Medical Oncology, Léon Bérard Cancer Center, Lyon, France; 4 Institut Universitaire du Cancer Toulouse Oncopôle, Toulouse, France; 5 Institut de Mathématiques de Toulouse, Université de Toulouse, Centre National de la Recherche Scientifique, Institut National des Sciences Appliquées de Toulouse, Toulouse, France; 6 Assistance Publique des Hôpitaux de Paris, Hôpital Hôtel Dieu, Centre d’Épidémiologie Clinique, INSERM U1153, Paris, France; 7 Department of Medical Oncology, Institut Claudius Regaud, Institut Universitaire du Cancer Toulouse, Toulouse, France

## Abstract

**Importance:**

Compared with standard cytotoxic therapies, randomized immune checkpoint inhibitor (ICI) phase 3 trials reveal delayed benefits in terms of patient survival and/or long-term response. Such outcomes generally violate the assumption of proportional hazards, and the classical Cox proportional hazards regression model is therefore unsuitable for these types of analyses.

**Objective:**

To evaluate the ability of the flexible parametric cure model (FPCM) to estimate treatment effects and long-term responder fractions (LRFs) independently of prespecified time points.

**Evidence Review:**

This systematic review used reconstructed individual patient data from ICI advanced or metastatic melanoma and lung cancer phase 3 trials extracted from the literature. Trials published between January 1, 2010, and October 1, 2019, with long-term follow-up periods (maximum follow-up, ≥36 months in first line and ≥30 months otherwise) were selected to identify LRFs. Individual patient data for progression-free survival were reconstructed from the published randomized ICI phase 3 trial results. The FPCM was applied to estimate treatment effects on the overall population and on the following components of the population: LRF and progression-free survival in non–long-term responders. Results obtained were compared with treatment effects estimated using the Cox proportional hazards regression model.

**Findings:**

In this systematic review, among the 23 comparisons studied using the FPCM, a statistically significant association between the time-to-event component and experimental treatment was observed in the main analyses and confirmed in the sensitivity analyses of 18 comparisons. Results were discordant for 4 comparisons that were not significant by the Cox proportional hazards regression model. The LRFs varied from 1.5% to 12.7% for the control arms and from 4.6% to 38.8% for the experimental arms. Differences in LRFs varied from 2% to 29% and were significantly increased in the experimental compared with the control arms, except for 4 comparisons.

**Conclusions and Relevance:**

This systematic review of reconstructed individual patient data found that the FPCM was a complementary approach that provided a comprehensive and pertinent evaluation of benefit and risk by assessing whether ICI treatment was associated with an increased probability of patients being long-term responders or with an improved progression-free survival in patients who were not long-term responders.

## Key Points

**Question:**

Does the flexible parametric cure model (FPCM) provide additional information compared with the classic Cox proportional hazards regression model in the analysis of randomized immune checkpoint inhibitor (ICI) clinical trials using progression-free survival as an end point?

**Findings:**

This systematic review of reconstructed individual patient data extracted from ICI advanced or metastatic melanoma and lung cancer phase 3 trials provides empirical evidence that FPCM is a complementary approach to the Cox proportional hazards regression model. The FPCM allows estimation of treatment effects on the overall population and on the following components of the population: long-term responder fraction and progression-free survival in non–long-term responders.

**Meaning:**

The findings of this review suggest that FPCM is a complementary approach that provides a comprehensive and pertinent evaluation of benefit and risk by assessing whether ICI treatment is associated with an increased probability of patients being long-term responders or with an improved progression-free survival in patients who are not long-term responders.

## Teaser

This systematic review assesses the ability of the flexible parametric cure model to determine whether immune checkpoint inhibitors are associated with an increased probability of being a long-term responder and improved progression-free survival in non–long-term responders.

## Introduction

Recent developments in immune checkpoint inhibitors (ICIs) have substantially improved the outcomes of patients with advanced and metastatic cancer across several different tumor types.^1,2,3,4^ Long-term analysis of the Keynote-001 (Study of Pembrolizumab [MK-3475] in Participants With Progressive Locally Advanced or Metastatic Carcinoma, Melanoma, or Non–Small Cell Lung Carcinoma) study^5^ of patients with advanced or metastatic non–small-cell lung cancer (NSCLC) receiving pembrolizumab reported a 5-year overall survival (OS) rate of 15.5%, a clinically meaningful improvement when compared with standard cytotoxic therapies.

Phase 3 trials^1,2^ comparing ICIs with standard therapies detect a delayed clinical effect of ICI treatments in terms of progression-free survival (PFS) and OS. Although survival curves for standard therapies and ICI overlap are sometimes even inverted early during follow-up, a clear separation between the 2 curves only becomes apparent several months after starting ICI treatment. The CheckMate-057 (Study of BMS-936558 [Nivolumab] Compared to Docetaxel in Previously Treated Metastatic Non-squamous NSCLC) study, for instance, found that patients with nonsquamous NSCLC treated with chemotherapy had better initial PFS compared with patients receiving ICIs.^2^ CheckMate-017 (Study of BMS-936558 [Nivolumab] Compared to Docetaxel in Previously Treated Advanced or Metastatic Squamous Cell NSCLC) similarly found that the PFS of patients with squamous NSCLC treated with nivolumab was identical to the initial 3 months of docetaxel treatment.^2^ The ICI treatments may nevertheless still provide durable responses and long-term PFS benefits compared with standard non-ICI agents.^6^ The presence of long-term responders is characterized by the appearance of subsequent plateaus in the survival curves as can be observed in patients with melanoma treated with ipilimumab and/or nivolumab^7^ and in patients with NSCLC treated with nivolumab.^2^ These contrasting observations must be considered when evaluating randomized clinical trials and highlight the challenges of randomized ICI trial analyses. When there is a delayed separation between survival curves and/or the presence of a plateau at the tail end of curves, the assumption of proportional hazards is generally violated, and the classic Cox proportional hazards regression model can no longer adequately quantify the effect size of the treatment.^8,9,10^

Several alternative approaches have been proposed and discussed to deal with nonproportional hazards,^11^ most notably the restricted mean survival time and the weighted log-rank test.^12,13^ The restricted mean survival time quantifies the effect of treatment, whereas the weighted log-rank test compares survival curves by allocating different weights to events, depending on the timing of the event. These approaches do not, however, allow for differentiation of whether ICIs increase the duration of the response. To address this question and accurately measure long-term treatment benefits, a previous study^14^ focused on analyzing the tail ends of survival curves using alternative approaches, such as milestone survival at prespecified time points. This approach consists of estimating PFS or OS rates and their corresponding CIs at specific time points using the Kaplan-Meier estimator. Milestone analysis has several drawbacks and needs to be interpreted with caution. It does not represent the entire survival curve but only captures information for a single time point. Moreover, PFS or OS estimates and their respective CIs may be misinterpreted when the length of the follow-up is inappropriate or indeed extends too far beyond the last observed event, when the risk set is small, and most patients have already been censored.^15^

To better understand the association of ICIs with treatment response and to provide a comprehensive and pertinent evaluation, the analysis must address 2 specific issues: (1) whether ICIs are associated with an increased probability of being a long-term responder and (2) whether ICIs are associated with an improved PFS in non–long-term responders. To date, various cure models for nonproportional hazards of long-term responders have been developed^16^ and applied to ICI trials.^17,18^ The current study evaluates the flexible parametric cure model (FPCM) on a representative data set of randomized clinical trials that compared nivolumab with docetaxel in previously treated patients with advanced NSCLC (CheckMate-057). This study also tested FPCM performances on advanced or metastatic melanomas and lung cancer data extracted from several randomized ICI phase 3 trials

## Methods

### Proof of Concept: CheckMate-057

**Figure 1.**
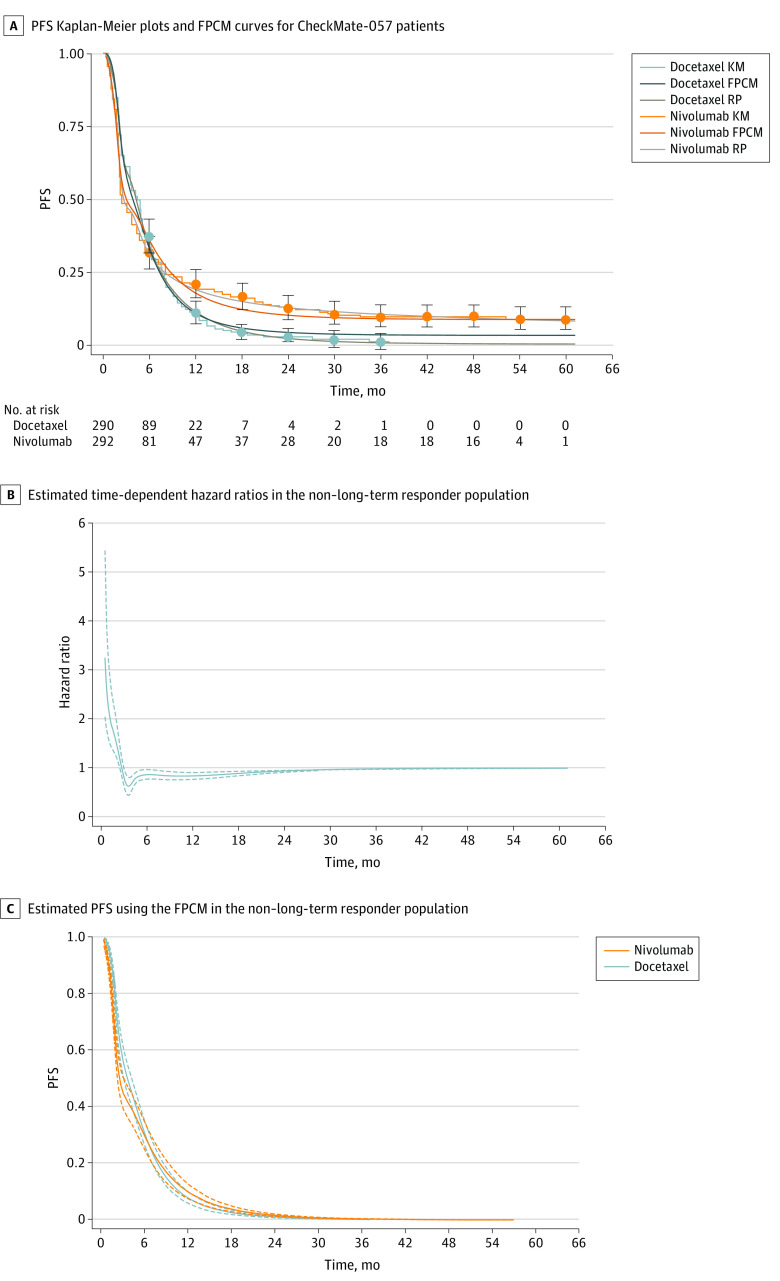
Progression-Free Survival (PFS) of the Study Patients A, Reconstructed PFS Kaplan-Meier (KM) plots and Flexible Parametric Cure Model (FPCM) curves for patients with non–small cell lung cancer in the CheckMate-057 study. B, Time-dependent hazard ratios with 95% CIs (dashed lines) estimated from the main analysis of the non–long-term responder population (RP). C, PFS and 95% CIs (dashed lines) estimated using FPCM in the non–long-term RP.

CheckMate- 057^19^was a randomized comparative phase 3 trial in patients with previously treated advanced nonsquamous NSCLCs. Patients were randomized to docetaxel (n = 290) and nivolumab (n = 292) treatment arms, and the published analyses^2,20^ of the trial report the crossover of PFS curves. The latest follow-up analysis^20^ detected no statistical difference in PFS between the 2 treatment arms using a classic Cox proportional hazards regression model (hazard ratio [HR], 0.89; 95% CI, 0.74-1.06). A nonnegligible long-term responder fraction (LRF) was observed in the nivolumab arm, with a 4-year PFS rate estimated at 9.6%. Updated PFS results were used as a representative data set for the PFS analysis (Figure 1A). In this study, a meta-analysis was not conducted because the main objective is to present a complementary approach to analyze ICI phase 3 trial data. Individual patient data (IPD) were reconstructed from published Kaplan-Meier curves for each trial arm using the iterative algorithm of Guyot et al.^21^

### Literature Search Strategy and Selection Criteria

**Figure 2.**
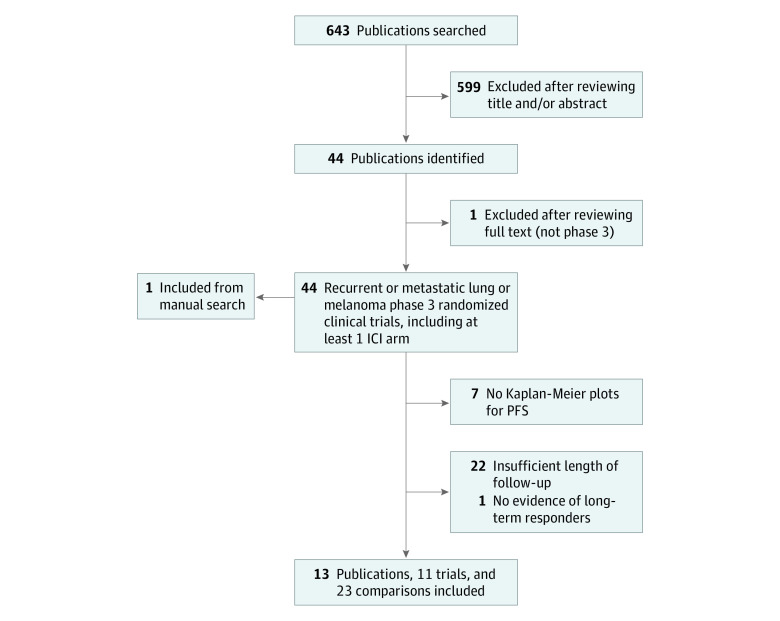
Selection Process of Immune Checkpoint Inhibitor (ICI) Randomized Phase 3 Trials From the Literature PFS indicates progression-free survival.

A PubMed literature search was conducted in October 2019 to identify phase 3 randomized clinical trial results published between January 1, 2010, and October 31, 2019, which included at least 1 recurrent and/or metastatic melanoma or lung cancer ICI arm and which evaluated phase 3 trials of preselected ICIs. Search strategy and selection criteria are detailed in eAppendix 1 (eTable 1) in the Supplement. As recommended in the statistical literature, trials with clinically insufficient follow-up periods and trials that lacked any clear clinical evidence of an LRF on estimated PFS curves were excluded.^22,23^ Among the 643 publications identified (Figure 2 and eAppendix 1 [eTables 2-4] in the Supplement), 13 publications corresponding to 11 clinical trials fulfilled the inclusion criteria (melanomas: 8 publications and 6 trials; NSCLCs: 5 publications and 5 trials).

### Data Extraction and IPD Reconstructions

The webplotdigitizer software, version 4.2 was used to extract the time and PFS coordinates from published curves.^24^ The number of at-risk patients and the number of events were extracted, if available. These data were then used as input in an iterative algorithm with Stata software, version 16 (StataCorp)^25^ that maps digitalized curves back to Kaplan-Meier data by finding numerical solutions to the inverted Kaplan-Meier equations. To validate IPD data reconstructions, we initially evaluated the accuracy of the algorithm (eAppendix 2 and eTable 5 in the Supplement).

### Statistical Analysis

The FPCMs were used to reanalyze the PFS data. In the flexible parametric survival model, restricted cubic splines with varying spline knots were used to model the log-cumulative hazard function over time, and a time-dependent treatment effect was investigated (eAppendix 3 in the Supplement). In an FPCM model, such as the Royston-Parmar model (RPM), the log-cumulative hazard function was given as follows:where is a restricted cubic spline function of log time with as the position of the knots and values for the parameters, is a treatment indicator, is the corresponding coefficient, and is a spline function for the time-dependent treatment effect with a vector of knots and values for the parameters. The RPM estimates time-dependent HRs and has been popularized for modeling treatment effects^26^ and as a supplementary analysis for randomized clinical trials.^27^

This model was adapted to estimate treatment effects and LRFs by forcing the log cumulative hazard in the flexible parametric survival model to plateau after the last knot. The cumulative hazard function was constrained to have a 0 slope by specifying knots in reverse order, and the last spline parameter was restricted to 0.^28^ The FPCMs are a special case of nonmixture cure models in which survival at time *t* can be written as (distribution F details are given in eAppendix 3 in the Supplement).^16^ The time-fixed component (constant parameters γ_00_ and β) is used to model the LRF, and covariates included in the time-dependent component (ie, distribution F) characterize a short-term effect. Parameters were estimated using maximum likelihood methods. The LRF treatment effect and short-term effect were tested using the Wald test. As proposed by Chen et al,^29^ survival of non–long-term responders was modeled as a function of LRF and distribution , which gave a time-dependent HR for non–long-term responders with corresponding 95% CIs (robust bootstrap method with 1000 samples).

The selection of the number of internal spline knots and spline knots for time-dependent effects was assessed using the bayesian information criterion (knot locations are presented in eTable 6 in the Supplement). We performed a sensitivity analysis to test the influence of the number of knots on the LRF estimation (eAppendix 4 in the Supplement). For trials with more than 2 arms (ie, >1 comparison), the FPCM was applied to each comparison. For each comparison, the goodness-of-fit was assessed by comparing the FPCM curves with the Kaplan-Meier estimates and the corresponding RPM curves (knot locations for RPM are presented in eTable 7 in the Supplement). The FPCM and RPM were fitted using the stpm2 module for flexible parametric survival models implemented in Stata.^28^ All statistical analyses were performed using Stata software, version 16 (StataCorp).

## Results

### Proof of Concept: CheckMate-057 Trial

Figure 1A presents Kaplan-Meier and best-fit FPCM and RPM PFS curves. Best-fit FPCM models comprised 5 internal knots and 1 internal knot for the baseline log-cumulative hazard and time-dependent effect, respectively. Visual inspection of the nivolumab and docetaxel arm FPCMs and Kaplan-Meier plots supported consistency, particularly because FPCM curves were contained within the 95% CIs of the Kaplan-Meier estimates (eFigure 1 in the Supplement). The RPM and FPCM present a similar fit for the tail of the distribution in the ICI arm. The PFS was lower for the RPM compared with the FPCM in the chemotherapy arm.

Although the classic Cox proportional hazards model detected no statistically significant difference between treatment (HR, 0.93; 95% CI, 0.77-1.11), the FPCM identified a treatment effect on both short-term PFS (time-dependent component *P* < .001) and the LRF (time-fixed component *P* < .001), with the HR remaining stable (HR, 0.54; 95% CI, 0.42-0.72) at 36 months, after initially decreasing from 5.69 to 0.49 at about 3 months (eFigure 2 in the Supplement). The LRFs were estimated at 3.0% (95% CI, 1.5%-5.3%) for the docetaxel arm and 8.5% (95% CI, 5.6%-12.0%) for the nivolumab arm. Nivolumab was therefore associated with a 5.4% (95% CI, 2.1%-8.8%) increase in the LRF compared with docetaxel. Results from sensitivity analyses were consistent (eAppendix 4 in the Supplement). A difference in the non–long-term responder population between arms over time (Figure 1B) and nivolumab PFS was lower during the first 6 months of treatment compared with docetaxel (Figure 1C).

### Characteristics of Selected Randomized Clinical Trials

Characteristics of the 11 clinical trials are presented in eTables 2 to 4 in the Supplement. The quality of data reconstruction is presented in eTable 5 in the Supplement and was deemed to be good. Data from 13 publications yielded 23 comparisons, with additional follow-up data for another 4 comparisons: 12 melanomas (2 comparisons performed 3 times [CheckMate-067 (Phase 3 Study of Nivolumab or Nivolumab Plus Ipilimumab Versus Ipilimumab Alone in Previously Untreated Advanced Melanoma)]) and 11 NSCLCs (2 comparisons performed twice [CheckMate-017 and CheckMate-057]) (Table).^1,2,7,20,30,31,32,33,34,35,36,37,38^

**Table.** Hazard Ratios Estimated Using the FPCM and the Classic Cox Proportional Hazards Regression Model

Trial	Experimental vs standard comparison	Hazard ratio (95% CI)		FPCM *P* value^b^		PFS of non–long-term responders in FPCM	Source
		Cox proportional hazards regression	FPCM^a^	LRF effect	Short-term effect		
**Melanoma: first-line treatment**							
CheckMate-066	Nivolumab vs dacarbazine	0.41 (0.32-0.52)	0.40 (0.32-0.51)	<.001	NA	SFT	Ascierto et, 2019^30^
CheckMate-067	Nivolumab vs ipilimumab	0.59 (0.49-0.71)	Time varying	<.001	<.001	SDE	Wolchok et al, 2017^31^
		0.56 (0.46-0.67)	Time varying	<.001	<.001	SDE	Hodi et al, 2018^7^
		0.61 (0.51-0.74)	Time varying	<.001	<.001	SDE	Larkin et al, 2019^32^
	Nivolumab plus ipilimumab vs ipilimumab alone	0.43 (0.35-0.53)	Time varying	<.001	<.001	SDE	Wolchok et al, 2017^31^
		0.40 (0.33-0.49)	0.40 (0.33-0.49)	<.001	NA	SFT	Hodi et al, 2018^7^
		0.41 (0.33-0.49)	0.41 (0.33-0.49)	<.001	NA	SFT	Larkin et al, 2019^32^
**Melanoma: first line or later**							
Intergroup trial E1690	10 mg/kg vs 3 mg/kg of ipilimumab	0.86 (0.74-1.01)	0.87 (0.75-1.02)	.09	NA	NS	Ascierto et al, 2017^33^
Keynote-006	Pembrolizumab every 2 weeks vs ipilimumab	0.57 (0.46-0.69)	0.57 (0.47-0.69)	<.001	NA	SFT	Robert et al, 2019^1^
	Pembrolizumab every 3 weeks vs ipilimumab	0.57 (0.47-0.70)	0.57 (0.47-0.70)	<.001	NA	SFT	
**Melanoma: second line or later**							
CheckMate-037	Nivolumab vs ICC	0.78 (0.59-1.02)	Time varying	.03	<.001	SDE	Larkin et al, 2018^34^
CA184-002	Ipilimumab plus GP100 vs GP100	0.85 (0.69-1.03)	0.84 (0.68-1.02)	.08	NA	SFT	Hodi et al, 2010^35^
**NSCLC: first-line treatment**							
CA184-104	Ipilimumab plus chemotherapy vs chemotherapy	0.90 (0.77-1.05)	0.90 (0.77-1.05)	.18	NA	NS	Govindan et al, 2017^36^
CheckMate-227 PDL1 ≥ 1%	Nivolumab plus ipilimumab vs chemotherapy	0.82 (0.70-0.98)	Time varying	<.001	<.001	SDE	Hellman et al, 2019^37^
	Nivolumab plus ipilimumab vs nivolumab	0.83 (0.71-0.98)	0.83 (0.71-0.98)	.02	NA	SFT	
CheckMate-227	Nivolumab plus ipilimumab vs chemotherapy	0.78 (0.61-0.99)	Time varying	<.001	<.001	SDE	
PDL1 < 1%	Nivolumab plus ipilimumab vs nivolumab plus chemotherapy	1.00 (0.79-1.27)	Time varying	.06	<.001	SDE	
	Nivolumab plus chemotherapy vs chemotherapy	0.71 (0.56-0.90)	0.72 (0.57-0.91)	.007	NA	SFT	
**NSCLC: second line**							
CheckMate-017	Nivolumab vs docetaxel	0.64 (0.49-0.84)	0.64 (0.49-0.83)	<.001	NA	SFT	Horn et al, 2017^2^
		0.65 (0.50-0.85)	Time varying	<.001	.009	SDE	Antonia et al, 2019^20^
CheckMate-057	Nivolumab vs docetaxel	0.92 (0.77-1.11)	Time varying	.004	<.001	SDE	Horn et al, 2017^2^
		0.93 (0.77-1.11)	Time varying	<.001	<.001	SDE	Antonia et al, 2019^20^
**NSCLC: second line or later**							
OAK	Atezolizumab vs docetaxel	0.98 (0.87-1.11)	Time varying	<.001	<.001	SDE	Fehrenbacher et al, 2018^38^

Abbreviations: CA184-002, MDX-010 Antibody, MDX-1379 Melanoma Vaccine, or MDX-010/MDX-1379 Combination Treatment for Patients With Unresectable or Metastatic Melanoma; CA184-104, Phase 3 Trial in Squamous Non Small Cell Lung Cancer Subjects Comparing Ipilimumab Vs Placebo in Addition to Paclitaxel and Carboplatin; CheckMate-017, Study of BMS-936558 [Nivolumab] Compared to Docetaxel in Previously Treated Advanced or Metastatic Squamous Cell NSCLC; CheckMate-037, A Study to Compare BMS-936558 to the Physician's Choice of Either Dacarbazine or Carboplatin and Paclitaxel in Advanced Melanoma Patients That Have Progressed Following Anti-CTLA-4 Therapy; CheckMate-057, Study of BMS-936558 [Nivolumab] Compared to Docetaxel in Previously Treated Metastatic Non-squamous NSCLC; CheckMate-066, Study of Nivolumab (BMS-936558) Compared With Dacarbazine in Untreated, Unresectable, or Metastatic Melanoma; CheckMate-067, Phase 3 Study of Nivolumab or Nivolumab Plus Ipilimumab Versus Ipilimumab Alone in Previously Untreated Advanced Melanoma; E1690, Phase 3 Trial in Subjects With Metastatic Melanoma Comparing 3 mg/kg Ipilimumab Versus 10 mg/kg Ipilimumab; FPCM, flexible parametric cure model; GP100, glycoprotein 100; ICC, investigator choice chemotherapy; Keynote-006, Study of Pembrolizumab [MK-3475] in Participants With Progressive Locally Advanced or Metastatic Carcinoma, Melanoma, or Non–Small Cell Lung Carcinoma; LRF, long-term responder fraction; NA, not applicable; NS, nonsignificant; NSCLC, non–small cell lung cancer; OAK, Study of Atezolizumab Compared With Docetaxel in Participants With Locally Advanced or Metastatic Non-Small Cell Lung Cancer Who Have Failed Platinum-Containing Therapy; PDL1, programmed cell death ligand 1; PFS, progression-free survival; SDE, significantly deleterious effect of the experimental treatment during early time points followed by a significant beneficial effect (direction of the effect varies over time); SFT, significantly in favor of the experimental treatment.

^a^ For models with time-dependent effects, a single hazard ratio may not provide a relevant measure of the treatment effect.

^b^ Treatment effect on the LRF was only tested for models with time-varying effects.

### Treatment Effect and Estimation of LRFs

**Figure 3.**
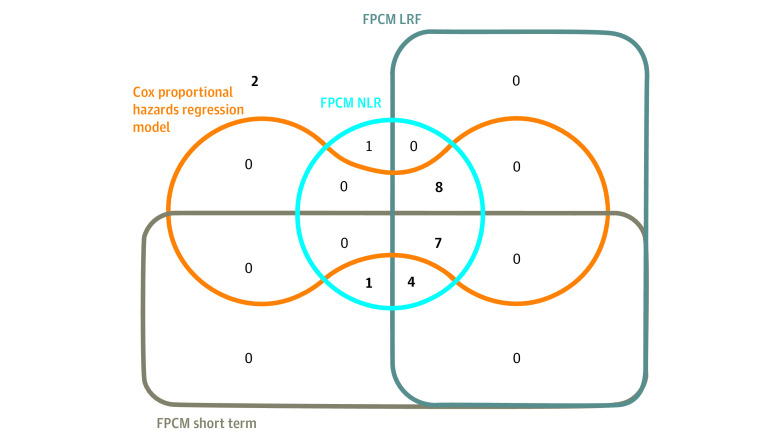
Overlaps of Significant Results Obtained With the Cox Proportional Hazards Model and With Individual Components of the Flexible Parametric Cure Model (FPCM) LRF indicates long-term responder fraction; NLR, non–long-term responders.

**Figure 4.**
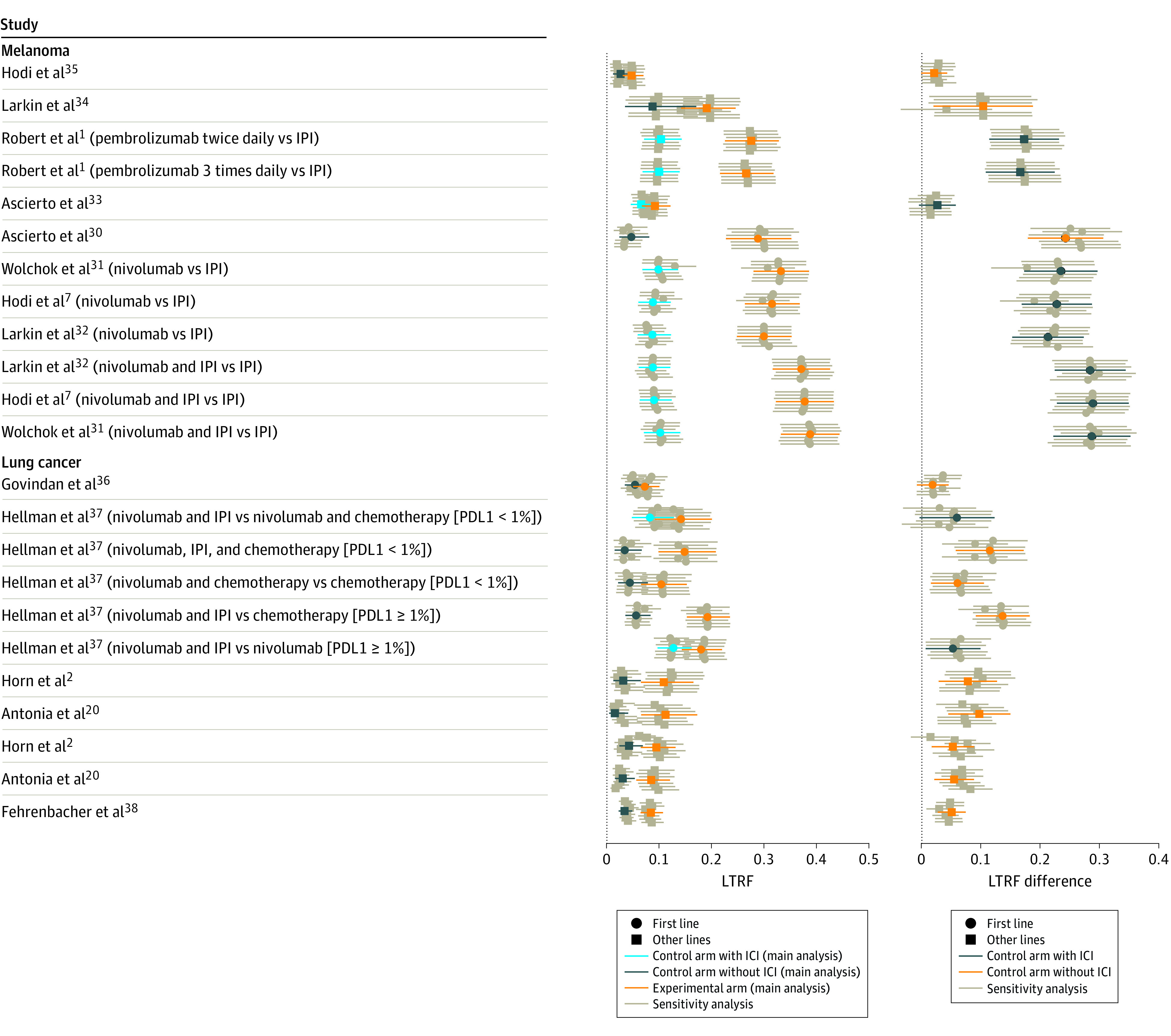
Long-term Responder Fraction (LTRF) and Rate of Long-term Response for the Standard and Experimental Arms In the center, comparison of LTRFs (95% CIs) in the different treatment arms. At right is the estimation of LTRF differences between the trial arms. IPI indicates immune checkpoint inhibitor; PDL1, programmed cell death ligand 1.

The main FPCM analysis is presented in eTables 8 and 9 and eFigures 3 and 4 in the Supplement. The HRs estimated using the Cox proportional hazards regression model and FPCM are reported in the Table. Figure 3 shows overlaps of the significant results obtained with the Cox proportional hazards regression model and with individual components of the FPCM. Using the FPCM, a time-dependent model was retained for 12 comparisons (melanoma: n = 5; NSCLC: n = 7). A statistically significant effect was identified on short-term PFS in all comparisons; for 11 comparisons (melanoma: n = 5; NSCLC: n = 6), we also identified an improvement in LRF. Among the 11 remaining comparisons, a treatment effect on LRF was identified by the FPCM for 8 comparisons (melanoma: n = 5; NSCLC: n = 3), with 3 comparisons showing no statistical differences between arms. Overall, results indicated a short-term effect on PFS for 12 comparisons (melanoma: n = 5; NSCLC: n = 7) and an LRF increase for 19 comparisons (melanoma: n = 10; NSCLC: n = 9). The Cox proportional hazards regression model did not find any statistically significant treatment effects for 8 comparisons (melanoma: n = 3; NSCLC: n = 5), whereas the FPCM retained a time-dependent treatment effect in 5 comparisons (melanoma: n = 1; NSCLC: n = 4). Statistically significant effects were observed on short-term PFS for all 5 comparisons and an increase in the LRF for 4 comparisons (melanoma: n = 1; NSCLC: n = 3). In the sensitivity analyses for knot location and number of knots, concordant results were observed for all comparisons except for 4, which yielded inconclusive results for short-term effect for 2 of them (eTable 10 and eFigure 5 in the Supplement). Figure 4A presents LRF estimates and their corresponding 95% CIs for standard and experimental arms. The rate of long-term responders varied from 1.5% (CheckMate-017^20^) to 12.7% (CheckMate-227 [An Investigational Immuno-therapy Trial of Nivolumab, or Nivolumab Plus Ipilimumab, or Nivolumab Plus Platinum-doublet Chemotherapy, Compared to Platinum Doublet Chemotherapy in Patients With Stage IV Non-Small Cell Lung Cancer]^37^) for the standard treatment arm and from 4.6% (CA184-002^35^ [MDX-010 Antibody, MDX-1379 Melanoma Vaccine, or MDX-010/MDX-1379 Combination Treatment for Patients With Unresectable or Metastatic Melanoma]) to 38.8% (CheckMate-067^31^) for the experimental arm. Figure 4B shows LRF differences with 95% CIs for each of the 23 comparisons. Differences in LRFs varied from 1.8% to 28.8% and were larger in melanoma (median, 22.0%, range, 2.1%-28.8%) compared with NSCLC (median, 5.9%; range, 1.8%-13.6%). In 18 (melanoma: n = 10; NSCLC: n = 8) of the 23 comparisons, LRFs of the experimental arm were at least twice that of the control arm. In sensitivity analyses, results were consistent for all comparisons (Figure 4A and B). Treatment arm PFS curves and time-dependent HRs in non–long-term responders are detailed in eFigures 6 and 7 in the Supplement. Visual assessment of graphs identified favorable experimental arm treatment effects for 9 comparisons (melanoma: n = 6; NSCLC: n = 3) (Table). Detrimental followed by beneficial experimental treatment effects were demonstrated for 12 comparisons (melanoma: n = 5; NSCLC: n = 7). No differences were observed over time for the remaining comparisons.

## Discussion

This systematic review supports that the FPCM may improve the analysis of randomized ICI clinical trials using PFS as an end point. When compared with the classic Cox proportional hazards regression model, the FPCM provides additional information for trials with complex survival patterns by incorporating a time-dependent HR estimation and/or taking into account LRFs. By extending the evaluation of treatment benefits to include a time scale and an LRF, the FPCM offers a direct clinically meaningful interpretation of treatment effects. The LRF component of the analysis, which refers to functional cure, is particularly relevant for patients.

Our findings specifically indicate that FPCM is a suitable strategy to fit randomized ICI phase 3 trial results and confirm that ICI treatments increase LRFs when compared with standard therapies. Considerable variations in the improvements of the LRFs, associated with the heterogeneity of clinical trial characteristics, were observed across trials. Melanomas have greater LRF increases compared with NSCLCs, irrespective of treatment lines. In melanomas, no significant differences were observed in the trial that included a population of highly pretreated patients^35^ and the phase 3 trial that compared the benefit-risk profile of 10 mg/kg vs 3 mg/kg of ipilimumab.^33^ The increase in the LRF was similar for the CheckMate-066 (Study of Nivolumab [BMS-936558] Compared With Dacarbazine in Untreated, Unresectable, or Metastatic Melanoma) trial (nivolumab vs dacarbazine) and for the ipilimumab vs nivolumab comparison in CheckMate-067. Analysis of 3 trials with at least 1 follow-up identified a nonnegligible fraction of long-term responders in the initial analysis. Notably, analysis of subsequent follow-up data indicated that these LRFs persisted over time and that the differences in LRFs between the different groups also remained constant. Indeed, accounting for follow-up data for the corresponding trials sometimes yielded different estimates of the drug’s benefit. Our results are consistent with reports from a previous study^39^ that introduced the concept of the functional curative potential of immunotherapy. Because our analysis focuses on the PFS end point, which is defined as disease progression or death, the *cure* term in FPCM is somewhat inappropriate because no one can actually be *cured* of death. However, in the interest of consistency with the literature, we will keep referring to our approach as FPCM.

The current study not only quantifies differences in the number of long-term responders in various treatment arms but also assesses the magnitude of long-term treatment benefits, which is particularly useful in the context of drug approvals where minimal clinically important differences between treatment arms have been predefined and where an experimental treatment may be considered clinically relevant if the observed difference is greater than this predefined threshold. Our FPCM approach also complements other systems that measure long-term benefits,^40,41^ specifically the American Society of Clinical Oncology Value Framework version 2 bonus and the European Society for Medical Oncology Value Framework, which both incorporate bonuses and adjustments that capture data from the tail ends of survival curves.^42,43^

The FPCMs have numerous advantages compared with other approaches that identify LRFs. Compared with milestone estimations, the FPCM examines the entire survival curve and does not require a previously specified meaningful survival milestone. However, use of the FPCM requires careful justification and mature data. Applying FPCM to estimate LRFs is warranted when there is enough supportive evidence from follow-up data to substantiate the identification of long-term responders and when survival plots exhibit tail-end plateaus. For any other cases, we recommend estimating treatment effects using a classic flexible parametric model without cure, instead of the FPCM.^12^ Because the FPCM is a classic flexible parametric model with a restriction on 1 of the parameters, it is comparable to a standard RPM for testing the assumption of cure. Because the formal test compares the fit over the whole time scale and not only the tail end that is used to estimate the cure proportion,^28^ it is recommended that the assumption of cure and the fit of the model be assessed visually from the graphs.^44^ Because no patient can be *cured* of death, analyses presented using the FPCM focus on PFS rather than OS, even though PFS is a secondary end point. Classic flexible parametric models without cure, such as the RPM,^12^ are better suited to evaluating treatment effects for OS end points.

### Limitations

The current study has a number of limitations. The study was not performed on original IPD but on reconstructed IPD. This type of approach has nevertheless been previously used by others^45,46^ and the accuracy of the reconstruction algorithm itself has been validated both in the literature^47^ and in our current analysis. When we compare results for trials with at least 1 follow-up, some discordant results may be associated with the quality of reconstructions. Our HRs were also estimated without adjusting or stratifying for randomization of stratification factors. Stratified analyses reduce bias when estimating treatment effects that violate the assumption of proportionality. Our study was also restricted to 23 FPCM comparisons of mature data because the length of follow-up of many of the trials retrieved from our initial PubMed searches was insufficient to identify long-term responders. Several comparisons involved late follow-ups of only a few patients, which may not allow sensible conclusions to be drawn because the interpretation of the Kaplan-Meier estimates may suffer from representativeness bias.^48^ For these comparisons, the RPM and FPCM have discrepancies at the tail ends of the distributions. A recent simulation study^49^ highlights that the FPCM may allow for the extrapolation of data and the corresponding LRF estimates may be accurate if the follow-up is sufficiently long. Finally, the FPCM needs to address several issues associated with to model selection^50^ and assessment schedule.^51^ The degree of complexity, dictated by the number and location of knots, needs to be balanced between goodness of fit and the risk of overfitting. For this reason, an automated process was used rather than a data-driven approach, and a sensitivity analysis was performed to evaluate the influence of model specification. The sensitivity analysis yielded concordant results for the short-term component of all but 3 of the comparisons. For these comparisons, the inconclusive results observed were in part related to the model selection process and tended to yield nonsignificant results. These inconclusive results concern only short-term PFS treatment effects and did not influence LRFs. As found in the sensitivity analysis, the number of knots and locations have little effect on the LRF result, which is pertinent for the patients. The oscillation observed in the time-dependent HR during the first few months after randomization may be associated not only with the assessment schedule but also with the hyperprogressive nature of the disease in ICI-treated patients.^6^

## Conclusions

The FPCM approach described in the current systematic review may have utility for both clinicians and health authorities to better describe treatment effects and estimate LRFs. This approach does not replace the classic Cox proportional hazards regression model for the primary analysis of randomized clinical trials but complements classic methods to evaluate treatment benefits. Because issues of delayed treatment effects and LRFs are not specific to ICI,^52^ an FPCM approach may be more widely applicable to clinical research to improve estimates of treatment benefits for other treatment strategies, such as targeted therapies. We suggest that clinicians and statisticians include an FPCM analysis in randomized phase 3 trial assessment to help regulatory agencies and clinicians evaluate the benefit-risk ratio of different therapies and to guide the selection of an optimal treatment strategy for individual patients. This FPCM approach may also help answer the fundamental question that all patients ask, “What is my probability of being functionally cured?”

## Notes

Supplement. **eAppendix 1.** Literature Searches **eAppendix 2.** Data Extraction and Accuracy of IPD Reconstruction **eAppendix 3.** Splines and Flexible Parametric Cure Model **eAppendix 4.** Royston and Parmar Model – Overall Survival **eAppendix 5.** Model Selection and Sensitivity Analysis **eTable 1.** Detailed Search Strategy **eTable 2.** Randomized Phase III Trials of Advanced/Metastatic Melanoma Identified From the Literature **eTable 3.** Randomized Phase III Trials of Advanced/Metastatic NSLC Identified From the Literature **eTable 4.** Randomized Phase III Trials Which Included Patients With Advanced/Metastatic SCLC Identified From Reviewing the Literature **eTable 5.** Reconstructed Data Quality After Digitalization (PFS) **eTable 6.** Internal Knot Locations for Baseline Hazard and Time-Dependent Treatment Effects (FPCM**)** **eTable 7.** Internal Knot Locations for Baseline Hazard and Time-Dependent Treatment Effects (Royston Parmar) **eTable 8.** Number of Internal Knots for Baseline Hazard (Κ_0_) and Time-Dependent Treatment Effects (Κ_1_) for the Main Analysis – Advanced/Metastatic Melanoma **eTable 9.** Number of Internal Knots for Baseline Hazard () and Time-Dependent Treatment Effects (Κ_1_) for the Main Analysis – Advanced/Metastatic NSCLC **eTable 10.** Estimation of Treatment Effects Using the FPCM and the Cox Proportional Model for the Main Analysis and Treatment Effects Obtained in the Sensitivity Analysis (6 Models) **eFigure 1.** Reconstructed PFS Kaplan-Meier Plots With 95% Confidence Intervals and FPCM Curves (Main Model) for Patients With Non-Small Cell Lung Cancer In Checkmate-057 **eFigure 2.** Time-Dependent Hazard Ratios With 95% Confidence Intervals Estimated From the Main Analysis Overall Population **eFigure 3.** Reconstructed PFS Kaplan-Meier Plots With 95% Confidence Intervals and FPCM Curves (Main Model) for Patients With Melanoma **eFigure 4.** Reconstructed PFS Kaplan-Meier Plots With 95% Confidence Intervals and FPCM Curves (Main Model) for Patients With Non-Small-Cell Lung Cancer **eFigure 5.** Checkmate 227 PDL1<1% Nivo+Ipi vs Nivo+ Chemo: Time-Dependent HRs With 95% Confidence Intervals Estimated From the Main and Sensitivity Analyses **eFigure 6.** Non-Long-Term Responder Population for patients with Melanoma **eFigure 7.** Non-Long-Term Responder Population for patients with NSCLC
