# Supplementary material for: Assessment of Treatment Effects and Long-term Benefits in Immune Checkpoint Inhibitor Trials Using the Flexible Parametric Cure Model: A Systematic Review
Source: JAMA Netw Open. 2021 Dec 21;4(12):e2139573. doi: 10.1001/jamanetworkopen.2021.39573 (PMC8693223; doi:10.1001/jamanetworkopen.2021.39573)
Supplement: Supplement. — eAppendix 1. Literature Searches eAppendix 2. Data Extraction and Accuracy of IPD Reconstruction eAppendix 3. Splines and Flexible Parametric Cure Model eAppendix 4. Royston and Parmar Model – Overall Survival eAppendix 5. Model Selection and Sensitivity Analysis eTable 1. Detailed Search Strategy eTable 2. Randomized Phase III Trials of Advanced/Metastatic Melanoma Identified From the Literature eTable 3. Randomized Phase III Trials of Advanced/Metastatic NSLC Identified From the Literature eTable 4. Randomized Phase III Trials Which Included Patients With Advanced/Metastatic SCLC Identified From Reviewing the Literature eTable 5. Reconstructed Data Quality After Digitalization (PFS) eTable 6. Internal Knot Locations for Baseline Hazard and Time-Dependent Treatment Effects (FPCM) eTable 7. Internal Knot Locations for Baseline Hazard and Time-Dependent Treatment Effects (Royston Parmar) eTable 8. Number of Internal Knots for Baseline Hazard (Κ0) and Time-Dependent Treatment Effects (Κ1) for the Main Analysis – Advanced/Metastatic Melanoma eTable 9. Number of Internal Knots for Baseline Hazard () and Time-Dependent Treatment Effects (Κ1) for the Main Analysis – Advanced/Metastatic NSCLC eTable 10. Estimation of Treatment Effects Using the FPCM and the Cox Proportional Model for the Main Analysis and Treatment Effects Obtained in the Sensitivity Analysis (6 Models) eFigure 1. Reconstructed PFS Kaplan-Meier Plots With 95% Confidence Intervals and FPCM Curves (Main Model) for Patients With Non-Small Cell Lung Cancer In Checkmate-057 eFigure 2. Time-Dependent Hazard Ratios With 95% Confidence Intervals Estimated From the Main Analysis Overall Population eFigure 3. Reconstructed PFS Kaplan-Meier Plots With 95% Confidence Intervals and FPCM Curves (Main Model) for Patients With Melanoma eFigure 4. Reconstructed PFS Kaplan-Meier Plots With 95% Confidence Intervals and FPCM Curves (Main Model) for Patients With Non-Small-Cell Lung Cancer eFigure 5. Checkmate 227 PDL1<1% Nivo [file jamanetwopen-e2139573-s001.pdf]

Supplementary Online Content

Filleron T, Bachelier M, Mazieres J, et al. Assessment of treatment effects and long-term benefits in immune checkpoint inhibitor trials using the flexible parametric cure model: a systematic review. *JAMA Netw Open*. 2021;4(12):e2139573. doi:10.1001/jamanetworkopen.2021.39573

eAppendix 1. Literature Searches
eAppendix 2. Data Extraction and Accuracy of IPD Reconstruction
eAppendix 3. Splines and Flexible Parametric Cure Model
eAppendix 4. Royston and Parmar Model – Overall Survival
eAppendix 5. Model Selection and Sensitivity Analysis
eTable 1. Detailed Search Strategy
eTable 2. Randomized Phase III Trials of Advanced/Metastatic Melanoma Identified From the Literature
eTable 3. Randomized Phase III Trials of Advanced/Metastatic NSLC Identified From the Literature
eTable 4. Randomized Phase III Trials Which Included Patients With Advanced/Metastatic SCLC Identified From Reviewing the Literature
eTable 5. Reconstructed Data Quality After Digitalization (PFS)
eTable 6. Internal Knot Locations for Baseline Hazard and Time-Dependent Treatment Effects (FPCM)
eTable 7. Internal Knot Locations for Baseline Hazard and Time-Dependent Treatment Effects (Royston Parmar)
eTable 8. Number of Internal Knots for Baseline Hazard ($\boldsymbol{K_0}$) and Time-Dependent Treatment Effects ($\boldsymbol{K_1}$) for the Main Analysis – Advanced/Metastatic Melanoma
eTable 9. Number of Internal Knots for Baseline Hazard (K_0) and Time-Dependent Treatment Effects (K_1) for the Main Analysis – Advanced/Metastatic NSCLC
eTable 10. Estimation of Treatment Effects Using the FPCM and the Cox Proportional Model for the Main Analysis and Treatment Effects Obtained in the Sensitivity Analysis (6 Models)
eFigure 1. Reconstructed PFS Kaplan-Meier Plots With 95% Confidence Intervals and FPCM Curves (Main Model) for Patients With Non-Small Cell Lung Cancer In Checkmate-057
eFigure 2. Time-Dependent Hazard Ratios With 95% Confidence Intervals Estimated From the Main Analysis Overall Population
eFigure 3. Reconstructed PFS Kaplan-Meier Plots With 95% Confidence Intervals and FPCM Curves (Main Model) for Patients With Melanoma
eFigure 4. Reconstructed PFS Kaplan-Meier Plots With 95% Confidence Intervals and FPCM Curves (Main Model) for Patients With Non-Small-Cell Lung Cancer
eFigure 5. Checkmate 227 PDL1<1% Nivo+Ipi vs Nivo+ Chemo: Time-Dependent HRs With 95% Confidence Intervals Estimated From the Main and Sensitivity Analyses
eFigure 6. Non-Long-Term Responder Population for patients with Melanoma
eFigure 7. Non-Long-Term Responder Population for patients with NSCLC

This supplementary material has been provided by the authors to give readers additional information about their work.

eAppendix 1. Literature Searches

PubMed search strategy

We focused on original English-language ICI articles, published between January 1, 2010 and October 1, 2019, which evaluated phase III trials of: atezolizumab, avelumab, durvalumab, ipilimumab, nivolumab, pembrolizumab and tremelimumab. Supplementary eTable 1 details PubMed search strategies performed in October 2019. TF and MB first reviewed all titles and abstracts to remove any obviously irrelevant publications and then examined the full-text articles to validate eligibility for inclusion in the current study. To ensure that no studies were missed, we also reviewed the references of original and review articles.

Reports of phase III randomized ICI trials in a recurrent and/or metastatic setting, with PFS as a (primary or secondary) endpoint, and which also included Kaplan-Meier survival curves in the publication were deemed eligible for inclusion in our current study. We excluded phase I, II or IV clinical trials, as well as phase III trials in adjuvant and neo-adjuvant settings. Subgroup analyses, meta-analyses and analyses using pooled data from two or more studies were also excluded.

eTable1. Detailed Search Strategy

Step	Search strategy	PubMed search string format
#1 AND	PubMed filter for articles indexed under disease category "neoplasms"	(neoplasms[MeSH Terms] OR Cancer OR Oncology)
#2 AND	Restrict to articles published in English	English [Language]
#3 AND	Restrict to articles published between January 2010 and September 2019	("2010/01/01"[Date - Entrez]: "2019/10/01"[Date - Entrez])
#4 AND	Restrict to phase III trials	("Randomized Controlled Trial"[Publication Type] NOT "clinical trial, phase ii"[Publication Type]) OR “Phase 3” [Title/Abstract] OR “Phase iii” [Title/Abstract] OR “Phase 3” [Title/Abstract])
#5 AND	Restrict to ICIs evaluated in phase III trials at the time of the current studywas conducted i.e., atezolizumab, avelumab, durvalumab, ipilimumab, nivolumab, pembrolizumab and tremelimumab.	((Nivolumab [Title/Abstract] OR CheckMate [Title/Abstract]) OR (pembrolizumab [Title/Abstract] OR keynote [Title/Abstract]) OR Atezolizumab [Title/Abstract] OR Durvalumab [Title/Abstract] OR Tremelimumab [Title/Abstract] OR Ipilimumab [Title/Abstract] OR Avelumab [Title/Abstract] OR cemiplimab [Title/Abstract])

© 2021 Filleron T et al. *JAMA Network Open.*

Eligibility Criteria

Only reports of phase III randomized trials of ICIs in recurrent and/or metastatic settings, with PFS as an endpoint (either primary or secondary), and with Kaplan-Meier survival plots were eligible for inclusion in our current study.

Trials were excluded:

- if the follow-up time was clinically insufficient
  - First Line: Maximum follow-up $\leq$ 36 months
  - Second line: Maximum follow-up $\leq$ 30 months
- if there was no clear evidence of a long-term responder fraction from a medical point of view

Characteristics of selected randomized controlled trials

Of the 643 publications identified from PubMed, 599 were excluded after reviewing titles and abstracts. One additional publication was identified from references of original and review articles. Among the 44 publications retained for full-text evaluation, 13 met our selection criteria. These included 11 phase III trials. Publications and characteristics of clinical trials are presented as supplementary data (Appendix 1, eTable 2 to eTable 4).

© 2021 Filleron T et al. *JAMA Network Open.*

Melanoma Publications

Phase III trials identified by PubMed Search (n=16)

- Robert C, Ribas A, Schachter J, Arance A, Grob JJ, Mortier L, Daud A, Carlino MS, McNeil CM, Lotem M, Larkin JMG, Lorigan P, Neyns B, Blank CU, Petrella TM, Hamid O, Su SC, Krepler C, Ibrahim N, Long GV. Pembrolizumab versus ipilimumab in advanced melanoma (KEYNOTE-006): post-hoc 5-year results from an open-label, multicentre, randomised, controlled, phase 3 study. Lancet Oncol. 2019 Sep;20(9):1239-1251
- Long GV, Dummer R, Hamid O, Gajewski TF, Caglevic C, Dalle S, Arance A, Carlino MS, Grob JJ, Kim TM, Demidov L, Robert C, Larkin J, Anderson JR, Maleski J, Jones M, Diede SJ, Mitchell TC. Epacadostat plus pembrolizumab versus placebo plus pembrolizumab in patients with unresectable or metastatic melanoma (ECHO-301/KEYNOTE-252): a phase 3, randomised, double-blind study. Lancet Oncol. 2019 Aug;20(8):1083-1097.
- Ascierto PA, Long GV, Robert C, Brady B, Dutriaux C, Di Giacomo AM, Mortier L, Hassel JC, Rutkowski P, McNeil C, Kalinka-Warzocha E, Savage KJ, Hernberg MM, Lebbé C, Charles J, Mihalcioiu C, Chiarion-Sileni V, Mauch C, Cognetti F, Ny L, Arance A, Svane IM, Schadendorf D, Gogas H, Saci A, Jiang J, Rizzo J, Atkinson V. Survival Outcomes in Patients With Previously Untreated BRAF Wild-Type Advanced Melanoma Treated With Nivolumab Therapy: Three-Year Follow-up of a Randomized Phase 3 Trial. JAMA Oncol. 2019 Feb 1;5(2):187-194.
- Hodi FS, Chiarion-Sileni V, Gonzalez R, Grob JJ, Rutkowski P, Cowey CL, Lao CD, Schadendorf D, Wagstaff J, Dummer R, Ferrucci PF, Smylie M, Hill A, Hogg D, Marquez-Rodas I, Jiang J, Rizzo J, Larkin J, Wolchok JD. Nivolumab plus ipilimumab or nivolumab alone versus ipilimumab alone in advanced melanoma (CheckMate 067): 4-year outcomes of a multicentre, randomised, phase 3 trial. Lancet Oncol. 2018 Nov;19(11):1480-1492.
- Larkin J, Minor D, D'Angelo S, Neyns B, Smylie M, Miller WH Jr, Gutzmer R, Linette G, Chmielowski B, Lao CD, Lorigan P, Grossmann K, Hassel JC, Sznol M, Daud A, Sosman J, Khushalani N, Schadendorf D, Hoeller C, Walker D, Kong G, Horak C, Weber J. Overall Survival in Patients With Advanced Melanoma Who Received Nivolumab Versus Investigator's Choice Chemotherapy in CheckMate 037: A Randomized, Controlled, Open-Label Phase III Trial. J Clin Oncol. 2018 Feb 1;36(4):383-390.
- Schachter J, Ribas A, Long GV, Arance A, Grob JJ, Mortier L, Daud A, Carlino MS, McNeil C, Lotem M, Larkin J, Lorigan P, Neyns B, Blank C, Petrella TM, Hamid O, Zhou H, Ebbinghaus S, Ibrahim N, Robert C. Pembrolizumab versus ipilimumab for advanced melanoma: final overall survival results of a multicentre, randomised, open-label phase 3 study (KEYNOTE-006). Lancet. 2017 Oct 21;390(10105):1853-1862.
- Wolchok JD, Chiarion-Sileni V, Gonzalez R, Rutkowski P, Grob JJ, Cowey CL, Lao CD, Wagstaff J, Schadendorf D, Ferrucci PF, Smylie M, Dummer R, Hill A, Hogg D, Haanen J, Carlino MS, Bechter O, Maio M, Marquez-Rodas I, Guidoboni M, McArthur G, Lebbé C, Ascierto PA, Long GV, Cebon J, Sosman J, Postow MA, Callahan MK, Walker D, Rollin L, Bhore R, Hodi FS, Larkin J. Overall Survival with Combined Nivolumab and Ipilimumab in Advanced Melanoma. N Engl J Med. 2017 Oct 5;377(14):1345-1356
- Ascierto PA, Del Vecchio M, Robert C, Mackiewicz A, Chiarion-Sileni V, Arance A, Lebbé C, Bastholt L, Hamid O, Rutkowski P, McNeil C, Garbe C, Loquai C, Dreno B, Thomas L, Grob JJ, Liszkay G, Nyakas M, Gutzmer R, Pikiel J, Grange F, Hoeller C, Ferraresi V, Smylie M, Schadendorf D, Mortier L, Svane IM, Hennicken D, Qureshi A, Maio M. Ipilimumab 10 mg/kg versus ipilimumab 3 mg/kg in patients with unresectable or metastatic melanoma: a randomised, double-blind, multicentre, phase 3 trial. Lancet Oncol. 2017 May;18(5):611-622
- Larkin J, Chiarion-Sileni V, Gonzalez R, Grob JJ, Cowey CL, Lao CD, Schadendorf D, Dummer R, Smylie M, Rutkowski P, Ferrucci PF, Hill A, Wagstaff J, Carlino MS, Haanen JB, Maio M, Marquez-Rodas I, McArthur GA, Ascierto PA, Long GV, Callahan MK, Postow MA, Grossmann K, Sznol M, Dreno B, Bastholt L, Yang A, Rollin LM, Horak C, Hodi FS, Wolchok JD. Combined Nivolumab and Ipilimumab or Monotherapy in Untreated Melanoma. N Engl J Med. 2015 Jul 2;373(1):23-34. doi: 10.1056/NEJMoa1504030. Epub 2015 May 31
- Robert C, Schachter J, Long GV, Arance A, Grob JJ, Mortier L, Daud A, Carlino MS, McNeil C, Lotem M, Larkin J, Lorigan P, Neyns B, Blank CU, Hamid O, Mateus C, Shapira-Frommer R, Kosh M, Zhou H, Ibrahim N, Ebbinghaus S, Ribas A; KEYNOTE-006 investigators. Pembrolizumab versus Ipilimumab in Advanced Melanoma. N Engl J Med. 2015 Jun 25;372(26):2521-3
- Maio M, Grob JJ, Aamdal S, Bondarenko I, Robert C, Thomas L, Garbe C, Chiarion-Sileni V, Testori A, Chen TT, Tschaika M, Wolchok JD. Five-year survival rates for treatment-naive patients with advanced melanoma who received ipilimumab plus dacarbazine in a phase III trial. J Clin Oncol. 2015 Apr 1;33(10):1191-6.
- Weber JS, D'Angelo SP, Minor D, Hodi FS, Gutzmer R, Neyns B, Hoeller C, Khushalani NI, Miller WH Jr, Lao CD, Linette GP, Thomas L, Lorigan P, Grossmann KF, Hassel JC, Maio M, Sznol M, Ascierto PA, Mohr P, Chmielowski B, Bryce A, Svane IM, Grob JJ, Krackhardt AM, Horak C, Lambert A, Yang AS, Larkin J. Nivolumab versus chemotherapy in patients with advanced melanoma who progressed after anti-

© 2021 Filleron T et al. *JAMA Network Open.*

CTLA-4 treatment (CheckMate 037): a randomised, controlled, open-label, phase 3 trial. Lancet Oncol. 2015 Apr;16(4):375-84

- Robert C, Long GV, Brady B, Dutriaux C, Maio M, Mortier L, Hassel JC, Rutkowski P, McNeil C, Kalinka-Warzocha E, Savage KJ, Hernberg MM, Lebbé C, Charles J, Mihalcioiu C, Chiarion-Sileni V, Mauch C, Cognetti F, Arance A, Schmidt H, Schadendorf D, Gogas H, Lundgren-Eriksson L, Horak C, Sharkey B, Waxman IM, Atkinson V, Ascierto PA. Nivolumab in previously untreated melanoma without BRAF mutation. N Engl J Med. 2015 Jan 22;372(4):320-30.
- Ribas A, Kefford R, Marshall MA, Punt CJ, Haanen JB, Marmol M, Garbe C, Gogas H, Schachter J, Linette G, Lorigan P, Kendra KL, Maio M, Trefzer U, Smylie M, McArthur GA, Dreno B, Nathan PD, Mackiewicz J, Kirkwood JM, Gomez-Navarro J, Huang B, Pavlov D, Hauschild A. Phase III randomized clinical trial comparing tremelimumab with standard-of-care chemotherapy in patients with advanced melanoma. J Clin Oncol. 2013 Feb 10;31(5):616-22.
- Robert C, Thomas L, Bondarenko I, O'Day S, Weber J, Garbe C, Lebbe C, Baurain JF, Testori A, Grob JJ, Davidson N, Richards J, Maio M, Hauschild A, Miller WH Jr, Gascon P, Lotem M, Harmankaya K, Ibrahim R, Francis S, Chen TT, Humphrey R, Hoos A, Wolchok JD. Ipilimumab plus dacarbazine for previously untreated metastatic melanoma. N Engl J Med. 2011 Jun 30;364(26):2517-26.
- Hodi FS, O'Day SJ, McDermott DF, Weber RW, Sosman JA, Haanen JB, Gonzalez R, Robert C, Schadendorf D, Hassel JC, Akerley W, van den Eertwegh AJ, Lutzky J, Lorigan P, Vaubel JM, Linette GP, Hogg D, Ottensmeier CH, Lebbé C, Peschel C, Quirt I, Clark JI, Wolchok JD, Weber JS, Tian J, Yellin MJ, Nichol GM, Hoos A, Urba WJ. Improved survival with ipilimumab in patients with metastatic melanoma. N Engl J Med. 2010 Aug 19;363(8):711-23.

Excluded after full-text review (not a phase III trial) (n=1)

- Postow MA, Chesney J, Pavlick AC, Robert C, Grossmann K, McDermott D, Linette GP, Meyer N, Giguere JK, Agarwala SS, Shaheen M, Ernstoff MS, Minor D, Salama AK, Taylor M, Ott PA, Rollin LM, Horak C, Gagnier P, Wolchok JD, Hodi FS. Nivolumab and ipilimumab versus ipilimumab in untreated melanoma. N Engl J Med. 2015 May 21;372(21):2006-17

Publication identified by manual search (n=1)

- Larkin J, Chiarion-Sileni V, Gonzalez R, Grob JJ, Rutkowski P, Lao CD, Cowey CL, Schadendorf D, Wagstaff J, Dummer R, Ferrucci PF, Smylie M, Hogg D, Hill A, Márquez-Rodas I, Haanen J, Guidoboni M, Maio M, Schöffski P, Carlino MS, Lebbé C, McArthur G, Ascierto PA, Daniels GA, Long GV, Bastholt L, Rizzo JI, Balogh A, Moshyk A, Hodi FS, Wolchok JD. Five-Year Survival with Combined Nivolumab and Ipilimumab in Advanced Melanoma. N Engl J Med. 2019

© 2021 Filleron T et al. *JAMA Network Open.*

Characteristics of clinical trials

eTable2. Randomized Phase III Trials of Advanced/Metastatic Melanoma Identified From the Literature

	Line	Experimental arm	Standard Arm	Reference		Comment
CA184-002<br>NCT00094653	2nd or latter	Ipilimumab + GP100	GP100<br>Ipilimumab	Hodi, NEJM 2010		
CA184-024<br>NCT00324155	1st	Ipilimumab+Dacarbazine	Dacarbazine+Placebo	Robert, NEJM 2011<br>Maio, JCO 2015		Insufficient follow-up<br>No PFS curves
NCT00257205	1st	Tremelimumab+SOC	SOC	Ribas, JCO 2013		No PFS curves
Checkmate-066<br>NCT01721772	1st	Nivolumab	Dacarbazine	Robert, NEJM 2015<br>Ascierto, JAMA Oncol 2019		Insufficient follow-up
Checkmate-037<br>NCT01721746	2nd	Nivolumab	ICC	Weber, Lancet Oncol 2015<br>Larkin, JCO 2018		No PFS curves of overall population
Keynote 006<br>NCT01866319	1st or 2nd	Pembrolizumab 10mg/kg Q2w<br>Pembrolizumab 10mg/kg Q3w	Ipilimumab	Robert, NEJM 2015<br>Schaster, Lancet 2017<br>Robert, Lancet Oncol 2019		Insufficient follow-up<br>Insufficient follow-up
CheckMate 067<br>NCT01844505	1st	Nivolumab<br>Nivolumab + Ipilimumab	Ipilimumab	Larkin, NEJM 2015<br>Wolchock, NEJM 2017<br>Hodi, Lancet 2018<br>Larkin, NEJM 2019		Insufficient follow-up
NCT01515189	1st or latter	Ipilimumab 10mg/kg	Ipilimumab 3mg/kg	Ascierto, Lancet Oncol 2017		
Keynote-252<br>NCT02752074	1st	Epacadostat + Pembrolizumab	Pembrolizumab	Long, Lancet Oncol 2019		Insufficient follow-up

© 2021 Filleron T et al. *JAMA Network Open.*

Lung Cancer Publications

Phase III trials identified from PubMed search (n=26)

- Antonia SJ, Borghaei H, Ramalingam SS, Horn L, De Castro Carpeño J, Pluzanski A, Burgio MA, Garassino M, Chow LQM, Gettinger S, Crinò L, Planchard D, Butts C, Drilon A, Wojcik-Tomaszewska J, Otterson GA, Agrawal S, Li A, Penrod JR, Brahmer J. Four-year survival with nivolumab in patients with previously treated advanced non-small-cell lung cancer: a pooled analysis. Lancet Oncol. 2019 Oct;20(10):1395-1408
- Hellmann MD, Paz-Ares L, Bernabe Caro R, Zurawski B, Kim SW, Carcereny Costa E, Park K, Alexandru A, Lupinacci L, de la Mora Jimenez E, Sakai H, Albert I, Vergnenegre A, Peters S, Syrigos K, Barlesi F, Reck M, Borghaei H, Brahmer JR, O'Byrne KJ, Geese WJ, Bhagavatheeswaran P, Rabindran SK, Kasinathan RS, Nathan FE, Ramalingam SS. Nivolumab plus Ipilimumab in Advanced Non-Small-Cell Lung Cancer. N Engl J Med. 2019 Sep 28. doi: 10.1056/NEJMoa1910231.
- West H, McCleod M, Hussein M, Morabito A, Rittmeyer A, Conter HJ, Kopp HG, Daniel D, McCune S, Mekhail T, Zer A, Reinmuth N, Sadiq A, Sandler A, Lin W, Ochi Lohmann T, Archer V, Wang L, Kowanetz M, Cappuzzo F. Atezolizumab in combination with carboplatin plus nab-paclitaxel chemotherapy compared with chemotherapy alone as first-line treatment for metastatic non-squamous non-small-cell lung cancer (IMpower130): a multicentre, randomised, open-label, phase 3 trial. Lancet Oncol. 2019 Jul;20(7):924-937
- Mok TSK, Wu YL, Kudaba I, Kowalski DM, Cho BC, Turna HZ, Castro G Jr, Srimuninnimit V, Laktionov KK, Bondarenko I, Kubota K, Lubiniecki GM, Zhang J, Kush D, Lopes G; KEYNOTE-042 Investigators. Pembrolizumab versus chemotherapy for previously untreated, PD-L1-expressing, locally advanced or metastatic non-small-cell lung cancer (KEYNOTE-042): a randomised, open-label, controlled, phase 3 trial. Lancet. 2019 May 4;393(10183):1819-1830
- Chih-Hsin Yang J, Shepherd FA, Kim DW, Lee GW, Lee JS, Chang GC, Lee SS, Wei YF, Lee YG, Laus G, Collins B, Pisetzky F, Horn L. Osimertinib Plus Durvalumab versus Osimertinib Monotherapy in EGFR T790M-Positive NSCLC following Previous EGFR TKI Therapy: CAURAL Brief Report. J Thorac Oncol. 2019 May;14(5):933-939.
- Wu YL, Lu S, Cheng Y, Zhou C, Wang J, Mok T, Zhang L, Tu HY, Wu L, Feng J, Zhang Y, Luft AV, Zhou J, Ma Z, Lu Y, Hu C, Shi Y, Baudelet C, Cai J, Chang J. Nivolumab Versus Docetaxel in a Predominantly Chinese Patient Population With Previously Treated Advanced NSCLC: CheckMate 078 Randomized Phase III Clinical Trial. J Thorac Oncol. 2019 May;14(5):867-875. doi: 10.1016/j.jtho.2019.01.006. Epub 2019 Jan 17.
- Reck M, Rodríguez-Abreu D, Robinson AG, Hui R, Csőszi T, Fülöp A, Gottfried M, Peled N, Tafreshi A, Cuffe S, O'Brien M, Rao S, Hotta K, Vandormael K, Riccio A, Yang J, Pietanza MC, Brahmer JR. Updated Analysis of KEYNOTE-024: Pembrolizumab Versus Platinum-Based Chemotherapy for Advanced Non-Small-Cell Lung Cancer With PD-L1 Tumor Proportion Score of 50% or Greater. J Clin Oncol. 2019 Mar 1;37(7):537-546. doi: 10.1200/JCO.18.00149. Epub 2019 Jan 8.
- von Pawel J, Bordoni R, Satouchi M, Fehrenbacher L, Cobo M, Han JY, Hida T, Moro-Sibilot D, Conkling P, Gandara DR, Rittmeyer A, Gandhi M, Yu W, Matheny C, Patel H, Sandler A, Ballinger M, Kowanetz M, Park K. Long-term survival in patients with advanced non-small-cell lung cancer treated with atezolizumab versus docetaxel: Results from the randomised phase III OAK study. Eur J Cancer. 2019 Jan;107:124-132. doi: 10.1016/j.ejca.2018.11.020. Epub 2018 Dec 17.
- Antonia SJ, Villegas A, Daniel D, Vicente D, Murakami S, Hui R, Kurata T, Chiappori A, Lee KH, de Wit M, Cho BC, Bourhaba M, Quantin X, Tokito T, Mekhail T, Planchard D, Kim YC, Karapetis CS, Hiret S, Ostoros G, Kubota K, Gray JE, Paz-Ares L, de Castro Carpeño J, Faivre-Finn C, Reck M, Vansteenkiste J, Spigel DR, Wadsworth C, Melillo G, Taboada M, Dennis PA, Özgüroğlu M; PACIFIC Investigators. Overall Survival with Durvalumab after Chemoradiotherapy in Stage III NSCLC. N Engl J Med. 2018 Dec 13;379(24):2342-2350.
- Horn L, Mansfield AS, Szczęsna A, Havel L, Krzakowski M, Hochmair MJ, Huemer F, Losonczy G, Johnson ML, Nishio M, Reck M, Mok T, Lam S, Shames DS, Liu J, Ding B, Lopez-Chavez A, Kabbinavar F, Lin W, Sandler A, Liu SV; IMpower133 Study Group. First-Line Atezolizumab plus Chemotherapy in Extensive-Stage Small-Cell Lung Cancer. N Engl J Med. 2018 Dec 6;379(23):2220-2229
- Paz-Ares L, Luft A, Vicente D, Tafreshi A, Gümüş M, Mazières J, Hermes B, Çay Şenler F, Csőszi T, Fülöp A, Rodríguez-Cid J, Wilson J, Sugawara S, Kato T, Lee KH, Cheng Y, Novello S, Halmos B, Li X, Lubiniecki GM, Piperdi B, Kowalski DM; KEYNOTE-407 Investigators. Pembrolizumab plus Chemotherapy for Squamous Non-Small-Cell Lung Cancer. N Engl J Med. 2018 Nov 22;379(21):2040-2051. d
- Barlesi F, Vansteenkiste J, Spigel D, Ishii H, Garassino M, de Marinis F, Özgüroğlu M, Szczesna A, Polychronis A, Uslu R, Krzakowski M, Lee JS, Calabrò L, Arén Frontera O, Ellers-Lenz B, Bajars M, Ruisi M, Park K. Avelumab versus docetaxel in patients with platinum-treated advanced non-small-cell lung cancer (JAVELIN Lung 200): an open-label, randomised, phase 3 study. Lancet Oncol. 2018 Nov;19(11):1468-1479.
- Fehrenbacher L, von Pawel J, Park K, Rittmeyer A, Gandara DR, Ponce Aix S, Han JY, Gadgeel SM, Hida T, Cortinovis DL, Cobo M, Kowalski DM, De Marinis F, Gandhi M, Danner B, Matheny C, Kowanetz M, He P, Felizzi F, Patel H, Sandler A, Ballinger M, Barlesi F. Updated Efficacy Analysis Including Secondary

© 2021 Filleron T et al. *JAMA Network Open.*

Population Results for OAK: A Randomized Phase III Study of Atezolizumab versus Docetaxel in Patients with Previously Treated Advanced Non-Small Cell Lung Cancer. J Thorac Oncol. 2018 Aug;13(8):1156-1170.

- Socinski MA, Jotte RM, Cappuzzo F, Orlandi F, Stroyakovskiy D, Nogami N, Rodríguez-Abreu D, Moro-Sibilot D, Thomas CA, Barlesi F, Finley G, Kelsch C, Lee A, Coleman S, Deng Y, Shen Y, Kowanetz M, Lopez-Chavez A, Sandler A, Reck M; IMpower150 Study Group. Atezolizumab for First-Line Treatment of Metastatic Nonsquamous NSCLC. N Engl J Med. 2018 Jun 14;378(24):2288-2301
- Gandhi L, Rodríguez-Abreu D, Gadgeel S, Esteban E, Felip E, De Angelis F, Domine M, Clingan P, Hochmair MJ, Powell SF, Cheng SY, Bischoff HG, Peled N, Grossi F, Jennens RR, Reck M, Hui R, Garon EB, Boyer M, Rubio-Viqueira B, Novello S, Kurata T, Gray JE, Vida J, Wei Z, Yang J, Raftopoulos H, Pietanza MC, Garassino MC; KEYNOTE-189 Investigators. Pembrolizumab plus Chemotherapy in Metastatic Non-Small-Cell Lung Cancer. N Engl J Med. 2018 May 31;378(22):2078-2092.
- Hellmann MD, Ciuleanu TE, Pluzanski A, Lee JS, Otterson GA, Audigier-Valette C, Minenza E, Linardou H, Burgers S, Salman P, Borghaei H, Ramalingam SS, Brahmer J, Reck M, O'Byrne KJ, Geese WJ, Green G, Chang H, Szustakowski J, Bhagavatheeswaran P, Healey D, Fu Y, Nathan F, Paz-Ares L. Nivolumab plus Ipilimumab in Lung Cancer with a High Tumor Mutational Burden. N Engl J Med. 2018 May 31;378(22):2093-210
- Vokes EE, Ready N, Felip E, Horn L, Burgio MA, Antonia SJ, Arén Frontera O, Gettinger S, Holgado E, Spigel D, Waterhouse D, Domine M, Garassino M, Chow LQM, Blumenschein G Jr, Barlesi F, Coudert B, Gainor J, Arrieta O, Brahmer J, Butts C, Steins M, Geese WJ, Li A, Healey D, Crinò L. Nivolumab versus docetaxel in previously treated advanced non-small-cell lung cancer (CheckMate 017 and CheckMate 057): 3-year update and outcomes in patients with liver metastases. Ann Oncol. 2018 Apr 1;29(4):959-965.
- Horn L, Spigel DR, Vokes EE, Holgado E, Ready N, Steins M, Poddubskaya E, Borghaei H, Felip E, Paz-Ares L, Pluzanski A, Reckamp KL, Burgio MA, Kohlhäeufl M, Waterhouse D, Barlesi F, Antonia S, Arrieta O, Fayette J, Crinò L, Rizvi N, Reck M, Hellmann MD, Geese WJ, Li A, Blackwood-Chirchir A, Healey D, Brahmer J, Eberhardt WEE. Nivolumab Versus Docetaxel in Previously Treated Patients With Advanced Non-Small-Cell Lung Cancer: Two-Year Outcomes From Two Randomized, Open-Label, Phase III Trials (CheckMate 017 and CheckMate 057). J Clin Oncol. 2017 Dec 10;35(35):3924-3933
- Antonia SJ, Villegas A, Daniel D, Vicente D, Murakami S, Hui R, Yokoi T, Chiappori A, Lee KH, de Wit M, Cho BC, Bourhaba M, Quantin X, Tokito T, Mekhail T, Planchard D, Kim YC, Karapetis CS, Hiret S, Ostoros G, Kubota K, Gray JE, Paz-Ares L, de Castro Carpeño J, Wadsworth C, Melillo G, Jiang H, Huang Y, Dennis PA, Özgüroğlu M; PACIFIC Investigators. Durvalumab after Chemoradiotherapy in Stage III Non-Small-Cell Lung Cancer. N Engl J Med. 2017 Nov 16;377(20):1919-1929.
- Govindan R, Szczesna A, Ahn MJ, Schneider CP, Gonzalez Mella PF, Barlesi F, Han B, Ganea DE, Von Pawel J, Vladimirov V, Fadeeva N, Lee KH, Kurata T, Zhang L, Tamura T, Postmus PE, Jassem J, O'Byrne K, Kopit J, Li M, Tschaika M, Reck M. Phase III Trial of Ipilimumab Combined With Paclitaxel and Carboplatin in Advanced Squamous Non-Small-Cell Lung Cancer. J Clin Oncol. 2017 Oct 20;35(30):3449-345
- Carbone DP, Reck M, Paz-Ares L, Creelan B, Horn L, Steins M, Felip E, van den Heuvel MM, Ciuleanu TE, Badin F, Ready N, Hiltermann TJN, Nair S, Juergens R, Peters S, Minenza E, Wrangle JM, Rodriguez-Abreu D, Borghaei H, Blumenschein GR Jr, Villaruz LC, Havel L, Krejci J, Corral Jaime J, Chang H, Geese WJ, Bhagavatheeswaran P, Chen AC, Socinski MA; CheckMate 026 Investigators. First-Line Nivolumab in Stage IV or Recurrent Non-Small-Cell Lung Cancer. N Engl J Med. 2017 Jun 22;376(25):2415-2426
- Rittmeyer A, Barlesi F, Waterkamp D, Park K, Ciardiello F, von Pawel J, Gadgeel SM, Hida T, Kowalski DM, Dols MC, Cortinovis DL, Leach J, Polikoff J, Barrios C, Kabbinavar F, Frontera OA, De Marinis F, Turna H, Lee JS, Ballinger M, Kowanetz M, He P, Chen DS, Sandler A, Gandara DR; OAK Study Group. Atezolizumab versus docetaxel in patients with previously treated non-small-cell lung cancer (OAK): a phase 3, open-label, multicentre randomised controlled trial. Lancet. 2017 Jan 21;389(10066):255-265.
- Reck M, Rodríguez-Abreu D, Robinson AG, Hui R, Csőszi T, Fülöp A, Gottfried M, Peled N, Tafreshi A, Cuffe S, O'Brien M, Rao S, Hotta K, Leiby MA, Lubiniecki GM, Shentu Y, Rangwala R, Brahmer JR; KEYNOTE-024 Investigators. Pembrolizumab versus Chemotherapy for PD-L1-Positive Non-Small-Cell Lung Cancer. N Engl J Med. 2016 Nov 10;375(19):1823-1833.
- Reck M, Luft A, Szczesna A, Havel L, Kim SW, Akerley W, Pietanza MC, Wu YL, Zielinski C, Thomas M, Felip E, Gold K, Horn L, Aerts J, Nakagawa K, Lorigan P, Pieters A, Kong Sanchez T, Fairchild J, Spigel D. Phase III Randomized Trial of Ipilimumab Plus Etoposide and Platinum Versus Placebo Plus Etoposide and Platinum in Extensive-Stage Small-Cell Lung Cancer. J Clin Oncol. 2016 Nov 1;34(31):3740-3748. d
- Herbst RS, Baas P, Kim DW, Felip E, Pérez-Gracia JL, Han JY, Molina J, Kim JH, Arvis CD, Ahn MJ, Majem M, Fidler MJ, de Castro G Jr, Garrido M, Lubiniecki GM, Shentu Y, Im E, Dolled-Filhart M, Garon EB. Pembrolizumab versus docetaxel for previously treated, PD-L1-positive, advanced non-small-cell lung cancer (KEYNOTE-010): a randomised controlled trial. Lancet. 2016 Apr 9;387(10027):1540-1550.
- Borghaei H, Paz-Ares L, Horn L, Spigel DR, Steins M, Ready NE, Chow LQ, Vokes EE, Felip E, Holgado E, Barlesi F, Kohlhäufl M, Arrieta O, Burgio MA, Fayette J, Lena H, Poddubskaya E, Gerber DE, Gettinger SN, Rudin CM, Rizvi N, Crinò L, Blumenschein GR Jr, Antonia SJ, Dorange C, Harbison CT, Graf Finckenstein F, Brahmer JR. Nivolumab versus Docetaxel in Advanced Nonsquamous Non-Small-Cell Lung Cancer. N Engl J Med. 2015 Oct 22;373(17):1627-39

© 2021 Filleron T et al. *JAMA Network Open.*

- Brahmer J, Reckamp KL, Baas P, Crinò L, Eberhardt WE, Poddubskaya E, Antonia S, Pluzanski A, Vokes EE, Holgado E, Waterhouse D, Ready N, Gainor J, Arén Frontera O, Havel L, Steins M, Garassino MC, Aerts JG, Domine M, Paz-Ares L, Reck M, Baudelet C, Harbison CT, Lestini B, Spigel DR. Nivolumab versus Docetaxel in Advanced Squamous-Cell Non-Small-Cell Lung Cancer. N Engl J Med. 2015 Jul 9;373(2):123-35.

© 2021 Filleron T et al. *JAMA Network Open.*

Characteristics of clinical trials

eTable 3. Randomized Phase III Trials of Advanced/Metastatic NSLC Identified From the Literature

	**Line**	**Experimental arm**	**Standard Arm**			
CheckMate-017<br>NCT01642004	2nd	Nivolumab	Docetaxel	Brahmer J, NEJM 2015<br>Horn, JCO 2017<br>Vokes, Annal of Onco 2017<br>Antonia, Lancet Oncol 2019		Insufficient follow-up<br><br>No PFS curve
CheckMate-057<br>NCT01673867	2nd	Nivolumab	Docetaxel	Borghaei, NEJM 2015<br>Horn, JCO 2017<br>Vokes, Annal of Onco 2017<br>Antonia, Lancet Oncol 2019		Insufficient follow-up<br><br><br>No PFS curve
KEYNOTE-010<br>NCT01905657	2nd	Pembrolizumab 2mg/kg<br>Pembrolizumab 10mg/kg	Docetaxel	Herbst, Lancet 2016		Insufficient follow-up
Keynote 024<br>NCT02142738	1st	Pembrolizumab	platinum-based chemotherapy+/- maintenance (permetrexed)	Reck, NEJM 2016<br>Reck, JCO 2019		Insufficient follow-up<br>No PFS curve
OAK<br>NCT02008227	2nd or latter	Atezolizumab	Docetaxel	Rittmeyer, Lancet 2017<br>Fehrenbacher, JTO 2018<br>Von Pawel, EJC 2019		Insufficient follow-up<br>No PFS curve
CHECKMATE-026<br>NCT02041533	1st	Nivolumab	Platinum-based Chemotherapy+/- maintenance (permetrexed)	Carbone, NEJM 2017		Insufficient follow-up
CA184-104<br>NCT01285609	1st	Ipilimumab+Chemotherapy	Chemotherapy	Govindan, JCO 2017		
PACIFIC<br>NCT02125461	Maintenance	Durvalumab	Placebo	Antonia, NEJM 2017<br>Antonia, NEJM 2018		Insufficient follow-up<br>Insufficient follow-up
Keynote 407<br>NCT02775435	1st	Pembrolizumab+Chemotherapy	Chemotherapy	Paz Ares, NEJM 2018		Insufficient follow-up
CheckMate-227-TMB<br>NCT02477826	1st	Nivolumab+Ipilimumab	Platinum-based Chemotherapy+/- maintenance (permetrexed)	Hellmann, NEJM 2018		Insufficient follow-up
CheckMate-227-PDL1>=1%<br>NCT02477826	1st	Nivolumab+Ipilimumab	Chemotherapy<br>Nivolumab	Hellmann, NEJM 2019		
CheckMate-227-PDL1<1%<br>NCT02477826	1st	Nivolumab+Ipilimumab<br>Nivolumab+Chemotherapy	Chemotherapy<br>Nivolumab+ Platinum based chemotherapy	Hellmann, NEJM 2019		
Keynote-189<br>NCT02578680	1st	Pembrolizumab+Chemotherapy	Platinum based chemotherapy	Gandhi, NEJM 2018		Insufficient follow-up
Impower-150<br>NCT02366143	1st	Atezolizumab + Bevacizumab + Carboplatin + Paclitaxel	Bevacizumab + Carboplatin + Paclitaxel	Socinski, NEJM 2018		Insufficient follow-up
Javelin Lung 200	2nd	Avelumab	Docetaxel	Barlesi Lancet Onco 2018		Insufficient follow-up

© 2021 Filleron T et al. *JAMA Network Open.*

NCT02395172						
KEYNOTE-042<br>NCT02220894	1st	Pembrolizumab	Chemotherapy	Mok, Lancet 2019		Insufficient follow-up
CHECKMATE 078<br>NCT02613507	2nd	Nivolumab	Docetaxel	Wu, J Thorac Oncol 2019		Insufficient follow-up
CAURAL<br>NCT02454933	2nd	Osimertinib + Durvalumab	Osimertinib	Yang, J Thorac Oncol 2019		No PFS curve. Early termination
IMPOWER 130<br>NCT02367781	2nd	Atezolizumab+chemotherapy	Chemotherapy	West Lancet Oncol, 2019		Insufficient follow-up

eTable 4. Randomized Phase III Trials Which Included Patients With Advanced/Metastatic SCLC Identified From Reviewing the Literature

	**Line**	**Experimental arm**	**Standard Arm**			
CA184-156<br>NCT01450761		Ipilimumab+ etoposide+platinum	Etoposide+platinum	Reck, JCO 2016		No clear evidence of long-term response
IMpower133<br>NCT02763579		Atezolizumab+Chemotherapy	Chemotherapy	Horn, NEJM 2018		Insufficient follow-up

© 2021 Filleron T et al. *JAMA Network Open.*

eAppendix 2. Data Extraction and Accuracy of IPD Reconstruction

Data extraction and IPD reconstruction

Individual Patient Data (IPD) were reconstructed from the published Kaplan-Meier curves for each of the trial arms using the iterative Guyot et al. algorithm (Guyot, BMC Med Res Methodol. 2012). The webplotdigitizer software was used to extract the time and PFS coordinates from published curves (Ohatgi A. Accessed February 18, 2020. https://automeris.io/WebPlotDigitizer/). The number of at-risk patients and the number of events were extracted, if available. IPD were reconstructed from extracted data using the iterative Guyot et al. algorithm on Stata (ipdfc) (Wei Y, Stata J 20177).

Accuracy of IPD reconstruction

Progression free survival endpoint

To evaluate the accuracy of the reconstructed IPD data, we compared the number of events, median PFS and PFS rates reported in published articles for given timepoints, to the corresponding reconstructed values obtained.

The number of events was reported in 7 publications (19 treatment arms). We found a median difference of 3.0 events (Range: [0.0; 29.0], mean: 5.7 and 95%CI=[1.7; 9.6]). The median PFS periods were reported in 13 publications (39 treatment arms) with a median difference of 0.2 months (range: [0.0; 0.7], mean: 0.2 and 95%CI=[0.1: 0.3]) between the values reported in the article and the reconstructed data.

In the 9 publications (30 treatment arms) that reported PFS estimates at given timepoints, we detected a median difference of 0.3 (Range: [0.0; 1.5], mean: 0.5 and 95%CI=[0.3; 0.7]), with the survival probability scale ranging from 0% to 100%.

© 2021 Filleron T et al. *JAMA Network Open.*

eTable5. Reconstructed Data Quality After Digitalization (PFS)

			Number of events			Median PFS			PFS rates at timepoint t		
Ref	Trial	Arm	Publi.	Digit.		Publi.	Digit.		t	Publi.	Digit.
Hodi, NEJM 2010	CA184-002	Ipilimumab		123		2.86	3.04		3	57.7	57.5
		GP100		129		2.76	2.91		3	48.5	49.4
		Ipilimumab + GP100		365		2.76	2.98		3	49.1	48.0
Ascierto, Lancet Oncol 2017	NCT01515189	Ipilimumab 3mg/kg	330	323		2.8	2.8				
		Ipilimumab 10mg/kg	328	314		2.8	3.0				
Larkin, JCO 2018	Checkmate 037	ICC	74	77		3.7	3.9				
		Nivolumab	182	184		3.1	3.5				
Robert, Lancet Oncol 2019	Keynote 006	Ipilimumab		216		3.4	3.6		48		
		Pembrolizumab 10mg/kg Q3w		197		9.7	9.8		48	23	23.0
		Pembrolizumab 10mg/kg Q2w		197		8.4	9.0		48	23	24.0
Wolchok, NEJM 2017	Checkmate 067	Ipilimumab		242		2.9	3.2		24	12	12.5
		Nivolumab + Ipilimumab		176		11.5	11.6		24	43	42.0
		Nivolumab		203		6.9	7.2		24	37	37.0
Hodi, Lancet Oncol 2018	Checkmate 067	Ipilimumab	258	253		2.9	3.0		48	9	9.7
		Nivolumab + Ipilimumab	182	177		11.5	12.1		48	37	37.2
		Nivolumab	201	203		6.9	7.2		48	31	31.1
Larkin, NEJM 2019	Checkmate 067	Ipilimumab		255		2.9	2.9		60	8	8.3
		Nivolumab + Ipilimumab		181		11.5	11.5		60	36	36.7
		Nivolumab		206		6.9	7.2		60	29	29.0
Ascierto, JAMA Oncol 2019	Checkmate 066	Dacarbazine	172	172		2.2	2.3		36	2.9	2.8
		Nivolumab	134	138		5.1	5.2		36	32.2	32.1
Horn, JCO 2017	Checkmate 017	Docetaxel	123	123		2.8	2.9		12	7	7.5
		Nivolumab	109	111		3.5	4.2		12	21	22.2
Antonia, Lancet Oncol 2019	Checkmate 017	Docetaxel	123	124		2.6	3.0		12	7.3	7.5
		Nivolumab	112	113		3.5	4.2		12	21.0	21.4
Horn, JCO 2017	Checkmate 057	Docetaxel	249	248		4.3	4.3		12	9	9.1
		Nivolumab	240	243		2.3	2.8		12	19	20.5
Antonia, Lancet Oncol 2019	Checkmate 057	Docetaxel	250	246		4.4	4.4		12	9.8	10.5
		Nivolumab	241	241		2.3	2.4		12	19.1	20.6
Fehrenbacher, JTO 2018	OAK	Docetaxel		530		3.8	4.0				
		Atezolizumab		538		2.7	2.8				
Govindan, JCO 2017	CA184-104	Placebo + Chemotherapy	345	316		5.6	5.6				
		Ipilimumab + Chemotherapy	359	334		5.6	5.6				
Hellmann, NEJM 2019	Checkmate 227 PDL1 ≥ 1%	Chemotherapy		282		5.6	5.6		12	19	18.9
		Nivolumab		312		4.2	4.2		12	26	26.2
		Nivolumab + Ipilimumab		285		5.0	5.0		12	33	34.1
Hellmann, NEJM 2019	Checkmate 227 PDL1 < 1%	Chemotherapy		144		4.7	5.2		12	14	13.9
		Nivolumab + Chemotherapy		144		5.6	5.8		12	26	25.8
		Nivolumab + Ipilimumab		134		5.1	5.1		12	31	31.9

Publi = From publication, Digit = from digitalized data

© 2021 Filleron T et al. *JAMA Network Open.*

eAppendix 3. Splines and Flexible Parametric Cure Model

Restricted cubic spline

The restricted cubic spline can be expressed as

$$s\{\ln(t), \boldsymbol{\gamma_0}, \boldsymbol{k_0}\} = \gamma_{00} + \gamma_{01}x_1(\ln(t)) + \cdots + \gamma_{0K-1}x_{K-1}(\ln(t))$$

with the derived functions $x_1, \ldots, x_K$ defined as follows

$$x_1(\ln(t)) = \ln(t)$$

$$x_j(\ln(t)) = \left(\ln(t) - k_{0j}\right)_+^3 - \theta_j(\ln(t) - k_{01})_+^3 - \left(1 - \theta_j\right)(\ln(t) - k_{0K})_+^3 \text{ for j=2,…,K-1}$$

Where $\theta_j = \frac{k_K - k_j}{k_K - k_1}$ and $(u)_+ = u$ if $u > 0$ and 0 if $u \le 0$.

Estimation of long-term responder fraction

Flexible parametric cure models were used to predict the long-term responder fraction by modeling the cumulative hazard (denoted H(t)). In a proportional hazards model, the log cumulative hazard function was modeled using natural cubic splines:

$$\ln\{H(t, z)\} = s\{\ln(t), \boldsymbol{\gamma_0}, \boldsymbol{k_0}\} + \beta z$$

where $s\{\ln(t), \boldsymbol{\gamma_0}, \boldsymbol{k_0}\}$ is the restricted cubic spline function of log time with $\boldsymbol{k_0} = (k_{01}, \ldots, k_{0K})$ the position of the K knots, $\boldsymbol{\gamma_0} = (\gamma_{00}, \ldots, \gamma_{0K-1})$ values for the parameters, z is the treatment and β the corresponding coefficient.

To deal with non-proportional hazards, a time-dependent effect was included in the model by including an interaction between treatment and a second spline function:

$$\ln\{H(t, z)\} = s\{\ln(t), \boldsymbol{\gamma_0}, \boldsymbol{k_0}\} + \beta z + s\{\ln(t), \boldsymbol{\gamma}_1, \boldsymbol{k_1}\}z$$

with $s\{\ln(t), \boldsymbol{\gamma}_1, \boldsymbol{k_1}\}$ the spline function for the time-dependent treatment effect with a vector of knots $\boldsymbol{k_1}$ and $\boldsymbol{\gamma}_1$ values for the parameters.

By adapting the Mozumder *et al.* approach (Mozumder et al., Statistics in Medicine 2018), the long-term responder fraction was estimated within the flexible parametric model by forcing the log cumulative hazard to plateau after the last knot (the cumulative hazard function was constrained toa zero slope after a specific timepoint). The knots were specified in reverse order $(k_K, \ldots, k_1)$ and the last spline parameter was restricted to zero. The cumulative hazard is then expressed as

$$H(t, z) = \exp[s\{\ln(t), \boldsymbol{\gamma_0}, \boldsymbol{k_0}\} + \beta z + s\{\ln(t), \boldsymbol{\gamma}_1, \boldsymbol{k_1}\}z]$$

The survival ($S(t, z)$) is given by

$$\exp[-H(t, z)] = \exp\{-\exp[s\{\ln(t), \boldsymbol{\gamma_0}, \boldsymbol{k_0}\} + \beta z + s\{\ln(t), \boldsymbol{\gamma}_1, \boldsymbol{k_1}\}z]\}$$

$$= \exp\{-\exp(\gamma_{00} + \beta z)\exp[\gamma_{02}x_2(\ln(t)) + \cdots + \gamma_{0K-1}x_{K-1}(\ln(t)) + s\{\ln(t), \boldsymbol{\gamma}_1, \boldsymbol{k_1}\}z]\}$$

© 2021 Filleron T et al. *JAMA Network Open.*

and the long-term responder fraction can be estimated for any treatment modality by $\exp[-\exp(\gamma_{00}+\beta z)]$ (time-fixed component) . FPCM is a special case of a non-mixture cure model with the distribution function $\mathrm{F}(t,Z)=\exp[\gamma_{02}x_2(\ln(t))+\cdots+\gamma_{0K-1}x_{K-1}(\ln(t))+s\{\ln(t),\boldsymbol{\gamma}_1,\boldsymbol{k_1}\}z]$ (time-dependent component).

Covariates included in distribution F (time-dependent component) characterize a "short-term effect", but covariates do not describe the survival for those who are not long-term responders (Othus, Clinical cancer research 2012).
As long as no time-dependent effects are modelled, the FPCM can be written as a proportional hazards model. For models with time-dependent effects, the PH assumptions are violated and HRs may not provide a relevant summary measure of the treatment effect in Table 1. The PH assumption is not appropriate, for example, where the primary effect of a treatment is ultimately in the long-term responder fraction, with little difference in early outcome, nor would it be appropriate if an early difference in outcome did not translate into a difference in the long-term responder fraction.

Estimation of treatment effect in the non-long-term responder population

As proposed by Chen et al. (Chen, JASA 2012), the mathematical expression of progression free survival in the non-long-term responder population was modelled as a function of the long-term responder fraction and the distribution characterizing the short-term effect

$$S_{NLR}(t,z)=\frac{S(t,z)-\exp\left[-\exp(\gamma_{00}+\beta z)\right]}{1-\exp\left[-\exp(\gamma_{00}+\beta z)\right]}$$

Progression Free survival in the non-long term responder population was predicted using the Newton–Raphson algorithm. In the non-long-term responder fraction, the treatment effect was therefore measured by a time-dependent hazard ratio with corresponding 95% confidence intervals (obtained by a robust bootstrap method with 1000 samples). Time varying hazard ratios and their corresponding confidence intervals were interpreted from graphs.

© 2021 Filleron T et al. *JAMA Network Open.*

Goodness-of-fit and location of internal knots

According to recommendations provided in the literature (Ng, Diagnostic and Prognostic Research 2018), restricted cubic splines ranging from four to eight knots (i.e., two to six interior knots) were examined for the baseline hazard. As recommended by Andersson et al. (Andersson, Stata Journal 2012), the knots were by default located at different percentiles of the distribution of uncensored log event times (eTable) and the last knot was placed after the last observed follow-up time. As fewer knots were required for the time-dependent effect, the number of internal knots was restricted between 1 and 5 and internal knots were placed at equally distributed quantiles of the log of the uncensored event times. A total of 20 FPCMs were investigated for each treatment comparison. Goodness-of-fit was assessed using the Akaike Information Criterion (AIC) and the Bayesian Information Criterion (BIC). Models with the lowest AIC and BIC were considered to have the best fit. In cases where AIC and BIC were discordant, the BIC was considered. The BIC corresponds to the most parsimonious model with the lowest number of knots and was preferred in order to limit the risk of over-parametrization.

The table below presents internal knot locations according to centiles of the distribution of the uncensored log survival times for baseline hazard and time-dependent treatment effects.

eTable6. Internal Knot Locations for Baseline Hazard and Time-Dependent Treatment Effects (FPCM**)**

No. of Internal knots	Baseline Hazard (Percentiles)	Time-dependent effect (Percentiles)
1	Not applicable	50
2	50 95	33 67
3	33 67 95	25 50 75
4	25 50 75 95	20 40 60 80
5	20 40 60 80 95	17 33 50 67 83
6	17 33 50 67 83 95	Not applicable

© 2021 Filleron T et al. *JAMA Network Open.*

eAppendix 4. Royston and Parmar Model – Overall Survival

Royston Parmar model - Goodness-of-fit and location of internal knots

In the Royston Parmar model, the mathematical expression of the log cumulative hazard is similar to the FPCM (Appendix C):

$$\ln\{H(t,z)\} = s\{\ln(t), \boldsymbol{\gamma_0}, \boldsymbol{k_0}\} + \beta z + s\{\ln(t), \boldsymbol{\gamma}_1, \boldsymbol{k_1}\}z$$

To improve the stability of the fitted function, the boundary knots of the restricted cubic splines are located at the extremes of uncensored survival times. To allow flexibility (Royston, Stat Med 2002), restricted cubic splines ranging from one to six interior knots were examined to model the baseline hazard. For the time-dependent effect, the number of investigated internal knots varied between 1 and 5. Models with the lowest AIC and BIC were considered to have the best fit. In cases where AIC and BIC were discordant, the BIC was considered. The BIC corresponds to the most parsimonious model with the lowest number of knots and was preferred to limit the risk of over-parametrization. The table below presents internal knot locations for the baseline hazard and the time-dependent treatment effect.

eTable 7. Internal Knot Locations for Baseline Hazard and Time-Dependent Treatment Effects (Royston Parmar)

No. of Internal knots	Baseline Hazard (Percentiles)	Time-dependent effect (Percentiles)
1	50	50
2	33 67	33 67
3	25 50 75	25 50 75
4	20 40 60 80	20 40 60 80
5	17 33 50 67 83	17 33 50 67 83
6	14 29 43 57 71 86	Not applicable

© 2021 Filleron T et al. *JAMA Network Open.*

eAppendix 5. Model Selection and Sensitivity Analysis

To evaluate the sensitivity of the number of knots in the FPCM, we compared the FPCM model hazard ratios and long-term responder fractions of different knot positions and varying numbers of knots. The sensitivity analysis was performed on the 6 models with the lowest BIC.

© 2021 Filleron T et al. *JAMA Network Open.*

Illustrative data set: Checkmate 57 update (Antonia, Lancet Oncol 2019)

Model selected for main analysis

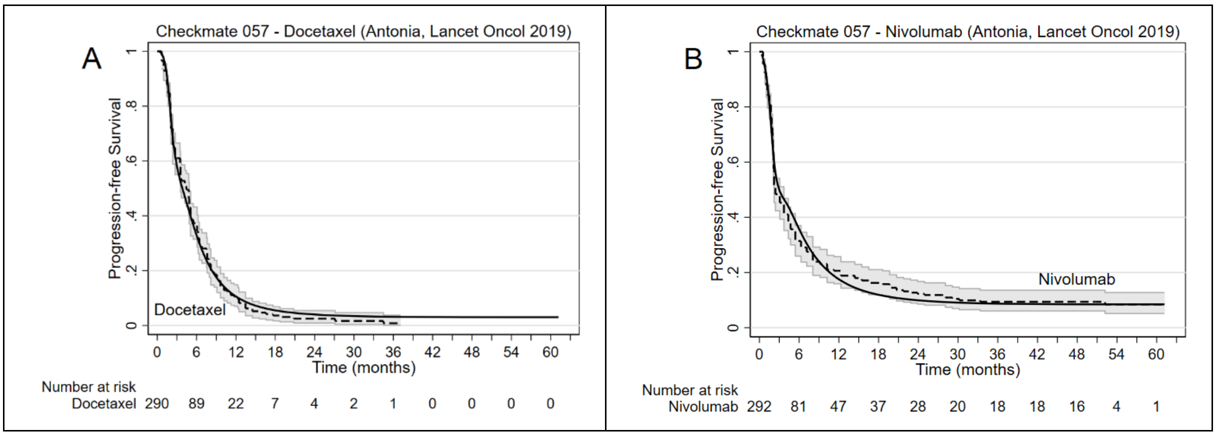

eFigure 1. Reconstructed PFS Kaplan-Meier Plots With 95% Confidence Intervals and FPCM Curves (Main Model) for Patients With Non-Small Cell Lung Cancer In Checkmate-057

A. Docetaxel Arm, B Nivolumab Arm.

Time varying hazard ratios estimated from main analysis: Overall

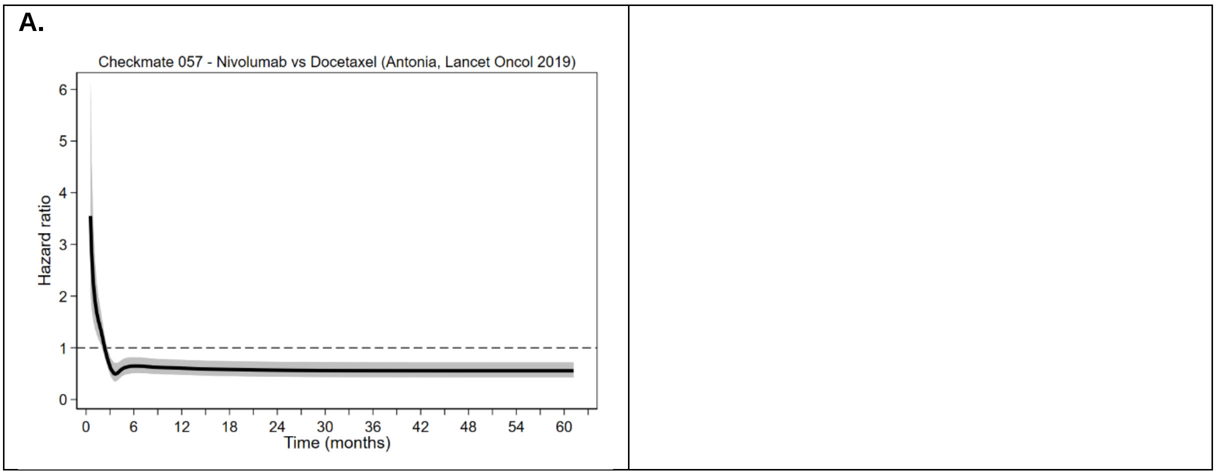

eFigure 2. Time-Dependent Hazard Ratios With 95% Confidence Intervals Estimated From the Main Analysis Overall Population

© 2021 Filleron T et al. *JAMA Network Open.*

Melanoma Trials

Model selected for main analysis

eTable8. Number of Internal Knots for Baseline Hazard ($\boldsymbol{K_0}$) and Time-Dependent Treatment Effects ($\boldsymbol{K_1}$) for the Main Analysis – Advanced/Metastatic Melanoma

Trial	Comparison			Main Model	
	Experimental vs Standard		**Ref**	K_0 / K_1	
		Melanoma Trial			
CA184-002	Ipilimumab + GP100 vs GP100		Hodi, NEJM 2010	5 / 0	
Checkmate066	Nivolumab vs Dacarbazine		Ascierto, JAMA Oncol 2019	6 / 0	
Checkmate037	Nivolumab vs ICC		Larkin, JCO 2018	5 / 4	
Keynote 006	Pembrolizumab Q2w vs Ipilimumab		Robert, Lancet Oncol 2019	5 / 0	
	Pembrolizumab Q3w vs Ipilimumab			5 / 0	
CheckMate-067	Nivolumab vs Ipilimumab		Wolchok, NEJM 2017	5 / 1	
			Hodi, Lancet Oncol 2018	6 / 1	
			Larkin, NEJM 2019	6 / 3	
	Nivolumab + Ipilimumab vs Ipilimumab		Wolchok, NEJM 2017	5 / 2	
			Hodi, Lancet Oncol 2018	5 / 0	
			Larkin, NEJM 2019	5 / 0	
NCT01515189	Ipilimumab 10mg/kg vs Ipilimumab 3mg/kg		Ascierto, Lancet Oncol 2017	6 / 0	

© 2021 Filleron T et al. *JAMA Network Open.*

Kaplan-Meier curves and FPCM (main analysis) based on reconstructed data

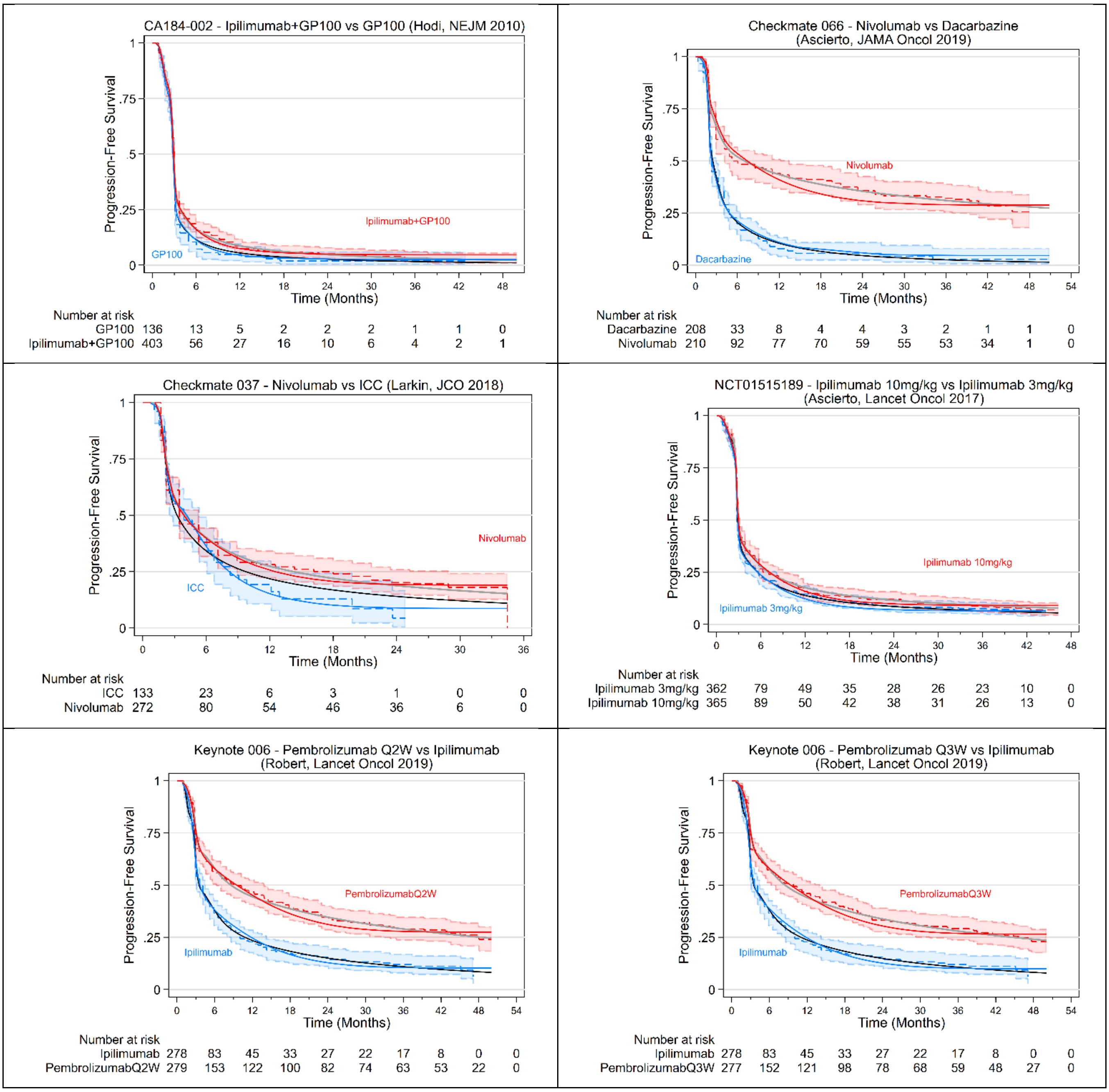

eFigure 3. Reconstructed PFS Kaplan-Meier Plots With 95% Confidence Intervals and FPCM Curves (Main Model) for Patients With Melanoma

© 2021 Filleron T et al. *JAMA Network Open.*

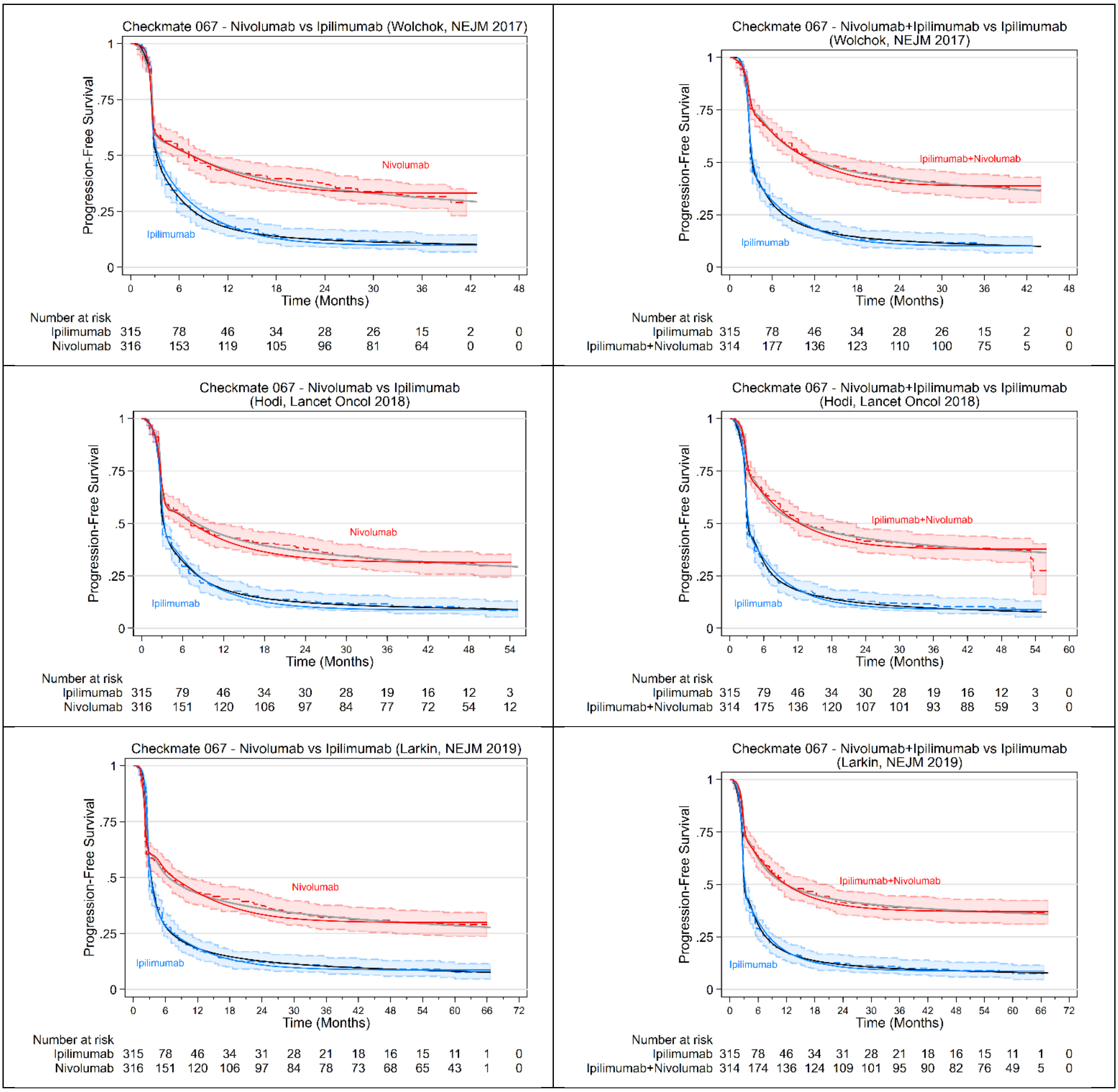

eFigure 3 (continued): Reconstructed PFS Kaplan-Meier plots with 95% confidence intervals and FPCM curves (main model) for patients with melanoma

© 2021 Filleron T et al. *JAMA Network Open.*

Non-small-cell lung cancer trials

Model selected for main analysis

eTable9. Number of Internal Knots for Baseline Hazard (K_0) and Time-Dependent Treatment Effects (K_1) for the Main Analysis – Advanced/Metastatic NSCLC

Trial	Comparison		Main Model	
	Experimental vs Standard	**Ref**	K_0 / K_1	
Checkmate 017	Nivolumab VS Docetaxel	Horn, JCO 2017	4 / 0	
		Antonia, Lancet Oncol 2019	5 / 1	
Checkmate 057	Nivolumab vs Docetaxel	Horn, JCO 2017	5 / 1	
		Antonia, Lancet Oncol 2019	5 / 1	
OAK	Atezolizumab vs Docetaxel	Fehrenbacher, JTO 2018	6 / 4	
CA184-104	Ipilimumab+Chemo. vs Chemotherapy	Govindan, JCO 2017	4 / 0	
Checkmate 227 PDL1≥1%	Nivolumab+Ipilimumab vs Chemotherapy	Hellmann, NEJM 2019	6 / 2	
	Nivolumab+Ipilimumab vs Nivolumab		6 / 0	
Checkmate 227 PDL1<1%	Nivolumab+Ipilimumab vs Chemotherapy	Hellmann, NEJM 2019	5 / 2	
	Nivolumab+Ipilimumab vs Nivolumab+Chemo		6 / 2	
	Nivolumab+ Chemotherapy vs Chemotherapy		6 / 0	

© 2021 Filleron T et al. *JAMA Network Open.*

Kaplan-Meier curves and FPCM (main analysis) based on reconstructed data

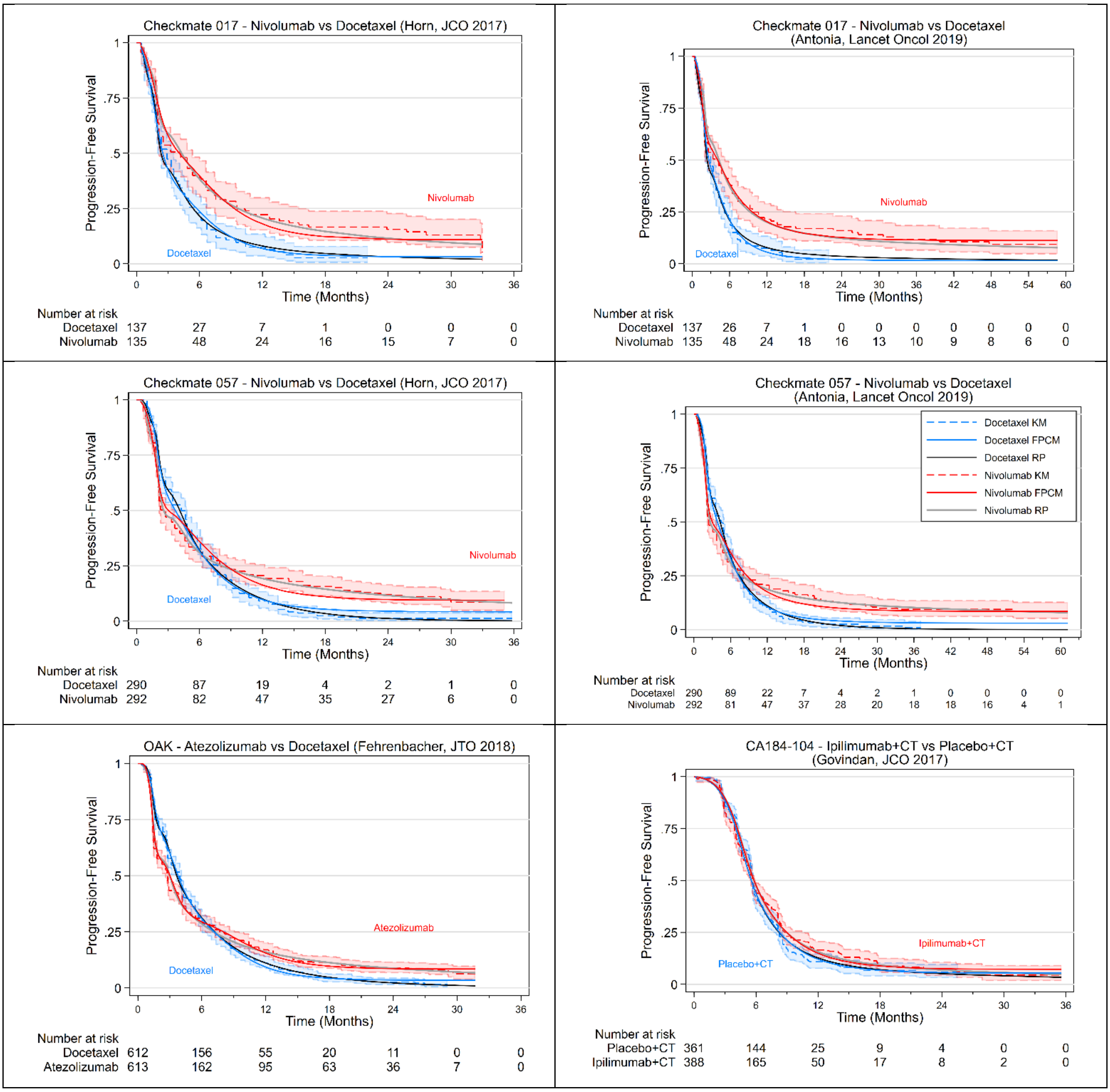

eFigure 4. Reconstructed PFS Kaplan-Meier Plots With 95% Confidence Intervals and FPCM Curves (Main Model) for Patients With Non-Small-Cell Lung Cancer

© 2021 Filleron T et al. *JAMA Network Open.*

Checkmate 227 - Nivolumab+Ipilimumab vs Chemotherapy
(Hellmann, NEJM 2019)

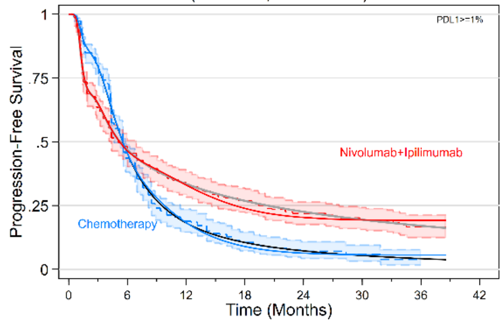

Number at risk								
Chemotherapy	397	131	44	23	12	8	1	0
Nivolumab+Ipilimumab	396	158	108	83	65	49	11	0

Checkmate 227 - Nivolumab+Ipilimumab vs Nivolumab
(Hellmann, NEJM 2019)

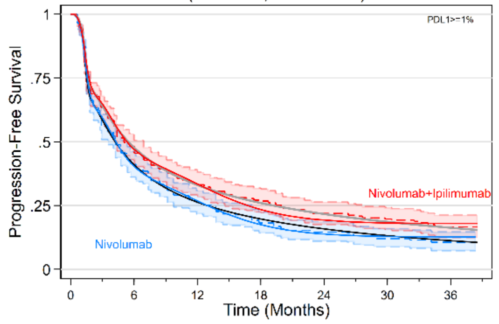

Number at risk							
Nivolumab	396	136	82	56	43	31	10
Nivolumab+Ipilimumab	396	158	108	83	65	49	11

Checkmate 227 - Nivolumab+Ipilimumab vs Chemotherapy
(Hellmann, NEJM 2019)

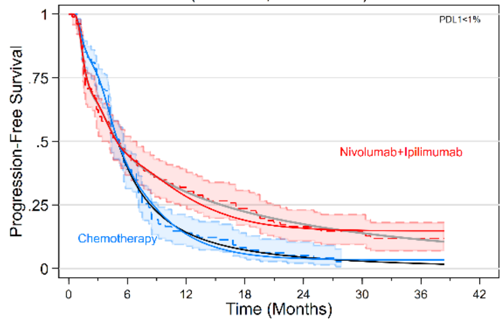

Number at risk								
Chemotherapy	186	58	16	8	5	0	0	0
Nivolumab+Ipilimumab	187	66	42	31	20	15	4	0

Checkmate 227 - Nivolumab+Ipilimumab vs Nivolumab+CT
(Hellmann, NEJM 2019)

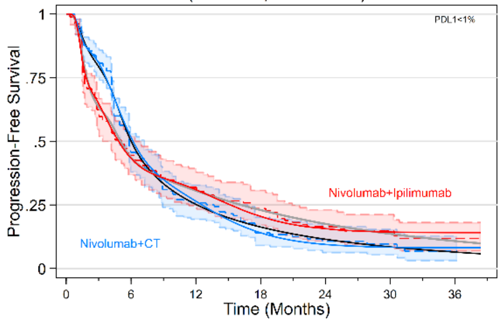

Number at risk							
Nivolumab+CT	177	73	37	19	11	7	1
Nivolumab+Ipilimumab	187	66	42	31	20	15	4

Checkmate 227 - Nivolumab+Ipilimumab vs Chemotherapy
(Hellmann, NEJM 2019)

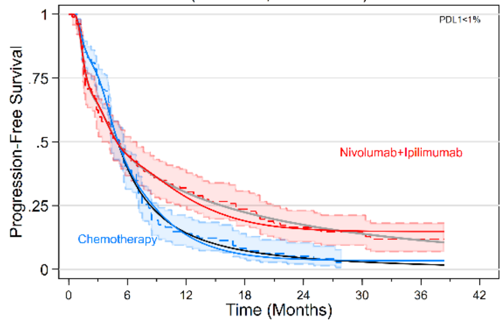

Number at risk								
Chemotherapy	186	58	16	8	5	0	0	0
Nivolumab+Ipilimumab	187	66	42	31	20	15	4	0

eFigure 4 (continued): Reconstructed PFS Kaplan-Meier plots with 95% confidence intervals and FPCM curves (main model) for patients with non-small-cell lung cancer

© 2021 Filleron T et al. *JAMA Network Open.*

Sensitivity Analysis

When the automatic process could not be performed, the sensitivity analysis of Checkmate-037 was conducted by manually assigning internal knots. The automatic knot finding process was not feasible when several knots were located at exactly the same timepoints. To avoid duplication of knot positions, one of the knots was therefore shifted to the next percentile of the event time.

Short-term treatment effect

The following table summarizes the sensitivity analysis and shows whether there is a statistically significant effect for each model: fixed effect and/or a time varying effect. For example, the models retained for the comparison of Nivo vs Ipi in Checkmate-067 (Wolchock, NEJM 2017) are: 1 model without tde (time-fixed component statistically significant) and 5 models with tde (both time-fixed and time-dependent component are statistically significant).

Discordant results between the main and the sensitivity analysis were observed for four comparisons of the time-dependent effect:

- CA184-104: 3 models without any significant time-fixed effect and 3 tde models with both components statistically significant. The FPCM retained for the main analysis is one of the most parsimonious.
- Checkmate 227 PDL1<1% (Nivo+Ipi vs Nivo+ Chemo.): Among the 6 models retained in the sensitivity analysis (all with time varying effects), only one was statistically significant for both time-fixed and time-dependent components, and 5 models were significant for the time-dependent component alone. **eFigure5 5** presents the time varying HR for the main and sensitivity analyses. The main model and the first model not retained in the sensitivity analysis (with 5 internal nodes for the log hazard and no nodes for the time-dependent effect) had similar BICs (main model BIC=1003; other model: 1010). The 1010 model was the most parsimonious compared to main and sensitivity models. This may explain the inconclusive results obtained for this particular trial.
- Checkmate-017 (Antonia, 2019): The main model contains a time-dependent effect (BIC:798.05). Among the 6 models retained in the sensitivity analysis, 4 models only had time-fixed effects and 2 models time-dependent effects. The first sensitivity model was more parsimonious compared to the main analysis (BIC=798.74). These results suggest only a treatment effect on the long-term responder fraction. The result obtained in one other comparison needs to be interpreted in details:
- Checkmate-067 (Nivo + Ipi vs Ipi) (Larkin, 2019): A short term effect was identified in four sensitivity models. The FPCM retained for the main analysis is the most parsimonious.

© 2021 Filleron T et al. *JAMA Network Open.*

eTable10. Estimation of Treatment Effects Using the FPCM and the Cox Proportional Model for the Main Analysis and Treatment Effects Obtained in the Sensitivity Analysis (6 Models)

Trial<br>Acrual period	Comparison		Main Analysis			Sensitivity Analysis (n=6) b			
	Experimental vs Standard	Ref<br>Cut-off date	Cox	FPCM a		Model with tve		LRF Significative	Short Term Significative
Melanoma Trial									
CA184-002	Ipi + GP100 vs GP100	Hodi, NEJM 2010	0.85 [0.69; 1.03]	0.84 [0.68; 1.02]	Tfc: p=0.082	5		0	0
Checkmate-066<br>2013/01 – 2014/02	Nivo (n=210) vs Dacarbazine (n=208)	Ascierto, JAMA Oncol 2019<br>2017/06	0.41 [0.32; 0.52]	0.40 [0.32; 0.51]	tfc: p <0.001	5		6	0
Checkmate-037<br>2012/12 - 2014/01	Nivo (n=272) vs ICC (n=133)	Larkin, JCO 2018<br>2016/03	0.78 [0.59; 1.02]	time varying	Tfc: p= 0.032<br>Tdc: p<0.001	5		5	0
Keynote-006<br>2013/09 - 2014/03	Pembro Q2w (n=279) vs Ipi (n=278)	Robert, Lancet Oncol 2019<br>2018/12	0.57 [0.46; 0.69]	0.57 [0.47; 0.69]	Tfc: p<0.001	4		6	0
	Pembro Q3w (n=277) vs Ipi (n=278)		0.57 [0.47; 0.70]	0.57 [0.47; 0.70]	Tfc: p<0.001	4		6	0
CheckMate-067<br>2013/07 - 2014/03	Nivo (n=316) vs Ipi (n=315)	Wolchok, NEJM 2017<br>2017/05	0.59 [0.49; 0.71]	time varying	Tfc: p<0.001<br>tdc p<0.001	5		6	5
		Hodi, Lancet Oncol 2018<br>2018/05	0.56 [0.46; 0.67]	time varying	Tfc: p<0.001<br>tdc: p<0.001	5		6	5
		Larkin, NEJM 2019<br>2019/07	0.61 [0.51; 0.74]	time varying	Tfc: p<0.001<br>tdc: p<0.001	6		6	6
	Nivo + Ipi (n=314) vs Ipi (n=315)	Wolchok, NEJM 2017<br>2017/05	0.43 [0.35; 0.53]	time varying	Tfc: p<0.001<br>tdc: p<0.001	4		6	3
		Hodi, Lancet Oncol 2018<br>2018/05	0.40 [0.33; 0.49]	0.40 [0.33; 0.49]	Tfc: p<0.001	5		6	0
		Larkin, NEJM 2019<br>2019/07	0.41 [0.33; 0.49]	0.41 [0.33;0.49]	Tfc: p<0.001	5		6	4
NCT01515189<br>2012/02 - 2012/07	Ipi 10mg/kg (n=365) vs Ipi 3mg/kg (n=362)	Ascierto, Lancet Oncol 2017	0.86 [0.74; 1.01]	0.87 [0.75; 1.02]	Tfc: p=0.092	5		0	0
Non-Small-Cell Lung Cancer									
Checkmate-017<br>2012/10 - 2013/12	Nivo (n=135) vs Docetaxel (n=137)	Horn, JCO 2017<br>2016/02	0.64 [0.49; 0.84]	0.64 [0.49; 0.83]	Tfc: p<0.001	4		6	0
		Antonia, Lancet Oncol 2019<br>2018/03	0.65 [0.50; 0.85]	time varying	Tfc: p<0.001<br>Tdc: p=0.009	2		6	2
Checkmate-057<br>2012/11 - 2013/12	Checkmate-057<br>2012/11 - 2013/12	Horn, JCO 2017<br>2016/02	0.92 [0.77; 1.11]	time varying	Tfc: p=0.004<br>Tdc: p<0.001	5		5	5
		Antonia, Lancet Oncol 2019	0.93 [0.77; 1.11]	time varying	Tfc: p<0.001	6		6	6

© 2021 Filleron T et al. *JAMA Network Open.*

		2018/03			Tdc: p<0.001				
OAK<br>2014/03 - 2015/04	Atezolizumab (n=613) vs Docetaxel (n=612)	Fehrenbacher, JTO 2018<br>2017/01	0.98 [0.87; 1.11]	time varying	Tfc: p<0.001<br>Tdc: p<0.001	6		6	6
CA184-104<br>2011/08 - 2015/06	Ipi+Chemo. (n=388) vs Chemo (n=361)	Govindan, JCO 2017<br>2015/09	0.90 [0.77; 1.05]	0.90 [0.77; 1.05]	Tfc: p=0.180	3		3	3
Checkmate-227<br>PDL1≥1%<br>2015/08 - 2016/11	Nivo+Ipi (n=396) vs Chemo (n=397)		0.82 [0.70; 0.98]	time varying	Tfc: p<0.001<br>Tdc: p<0.001	6		6	6
	Nivo+Ipi (n=396) vs Nivo (n=396)		0.83 [0.71; 0.98]	0.83 [0.71; 0.98]	Tfc: p=0.025	5		6	1
Checkmate-227<br>PDL1<1%	Nivo+Ipi (n=187) vs Chemo (n=186)	Hellmann, NEJM 2019<br>2019/07	0.78 [0.61; 0.99]	time varying	Tfc: p<0.001<br>Tdc: p<0.001	6		6	6
	Nivo+Ipi (n=187) vs Nivo+ Chemo (n=177)	Hellmann, NEJM 2019	1.00 [0.79; 1.27]	time varying	Tfc: p=0.063<br>Tdc:p<0.001	6		0	6
	Nivo+ Chemo. (n=177) vs Chemo (n=186)		0.71 [0.56; 0.90]	0.72 [0.57; 0.91]	Tfc: P=0.007	5		6	0

a For models with a time-dependent effect one single HR may not provide a relevant measure of the treatment effect.

b. Number of sensitivity analyses with a model with time varying effect, Number of sensitivity analyses with a significant treatment effect in the LRF, Number of sensitivity analyses with a significant short term treatment effect.

© 2021 Filleron T et al. *JAMA Network Open.*

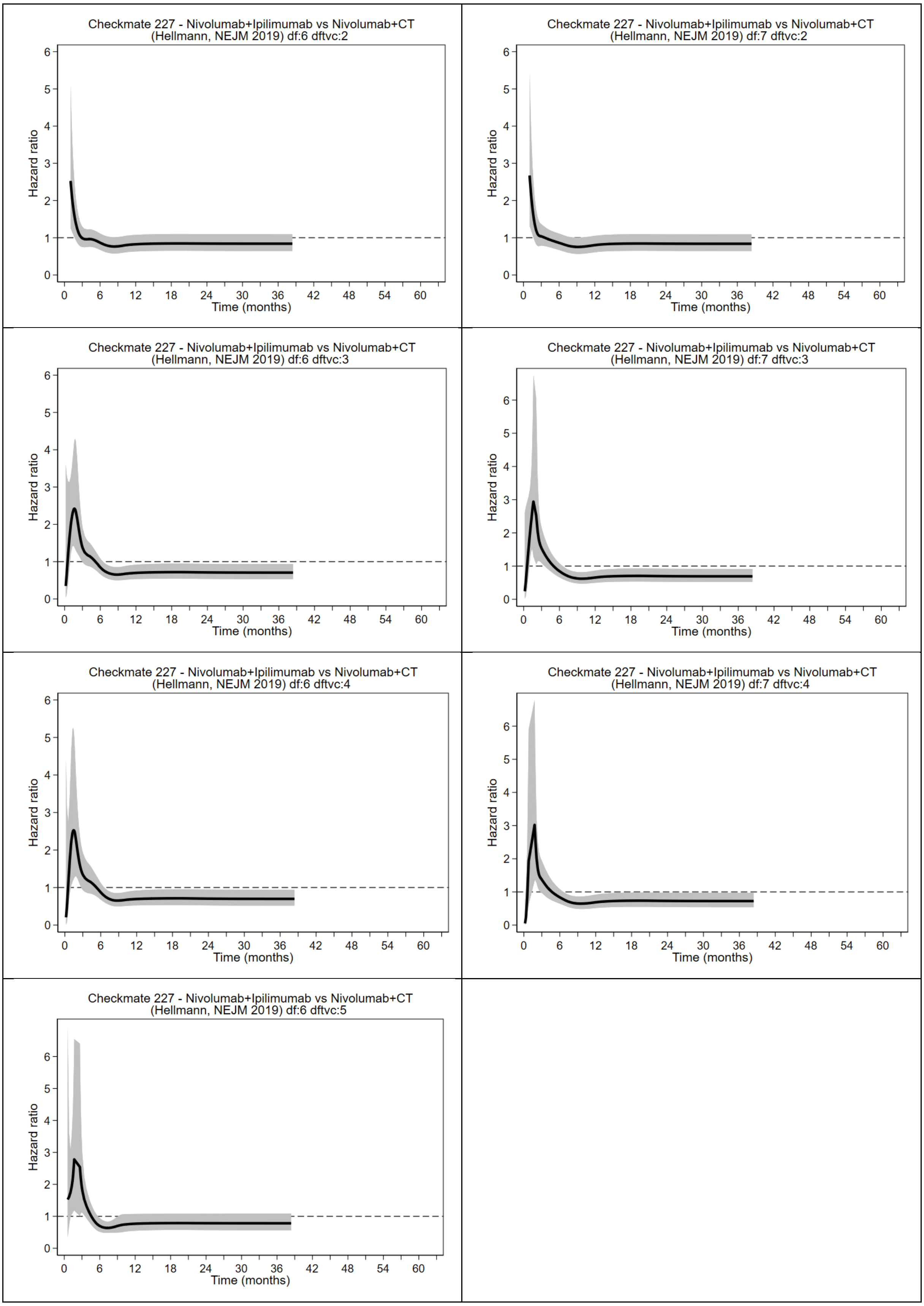

eFigure5. Checkmate 227 PDL1<1% Nivo+Ipi vs Nivo+ Chemo: Time-Dependent HRs With 95% Confidence Intervals Estimated From the Main and Sensitivity Analyses

© 2021 Filleron T et al. *JAMA Network Open.*

Progression free survival and treatment effect in the non-long-term responder population

Melanoma

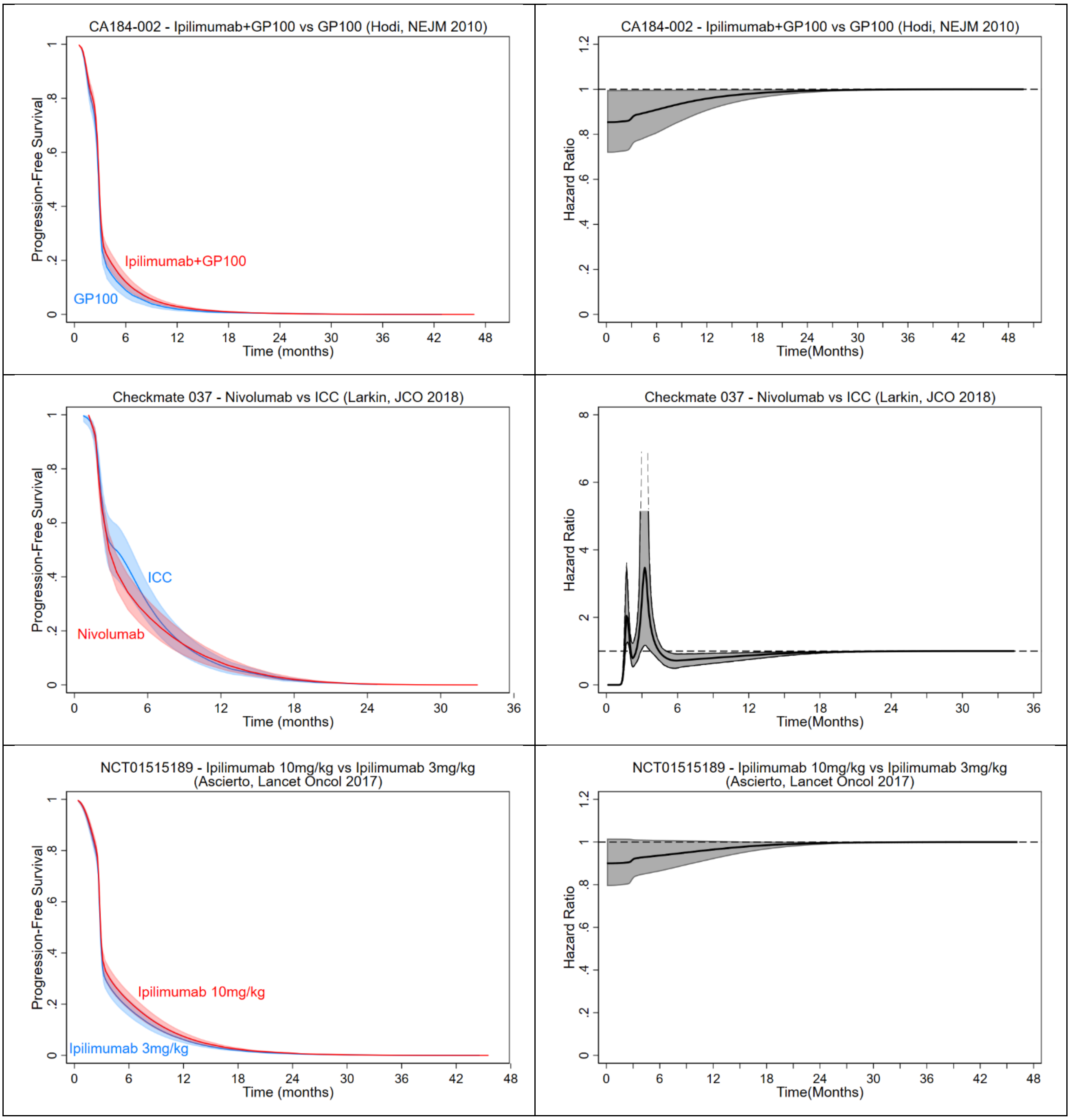

eFigure 6. Non-Long-Term Responder Population for patients with melanoma

A. Progression free survival B. Hazard ratio and 95% confidence intervals and FPCM curves (main model) for patients with melanoma

© 2021 Filleron T et al. *JAMA Network Open.*

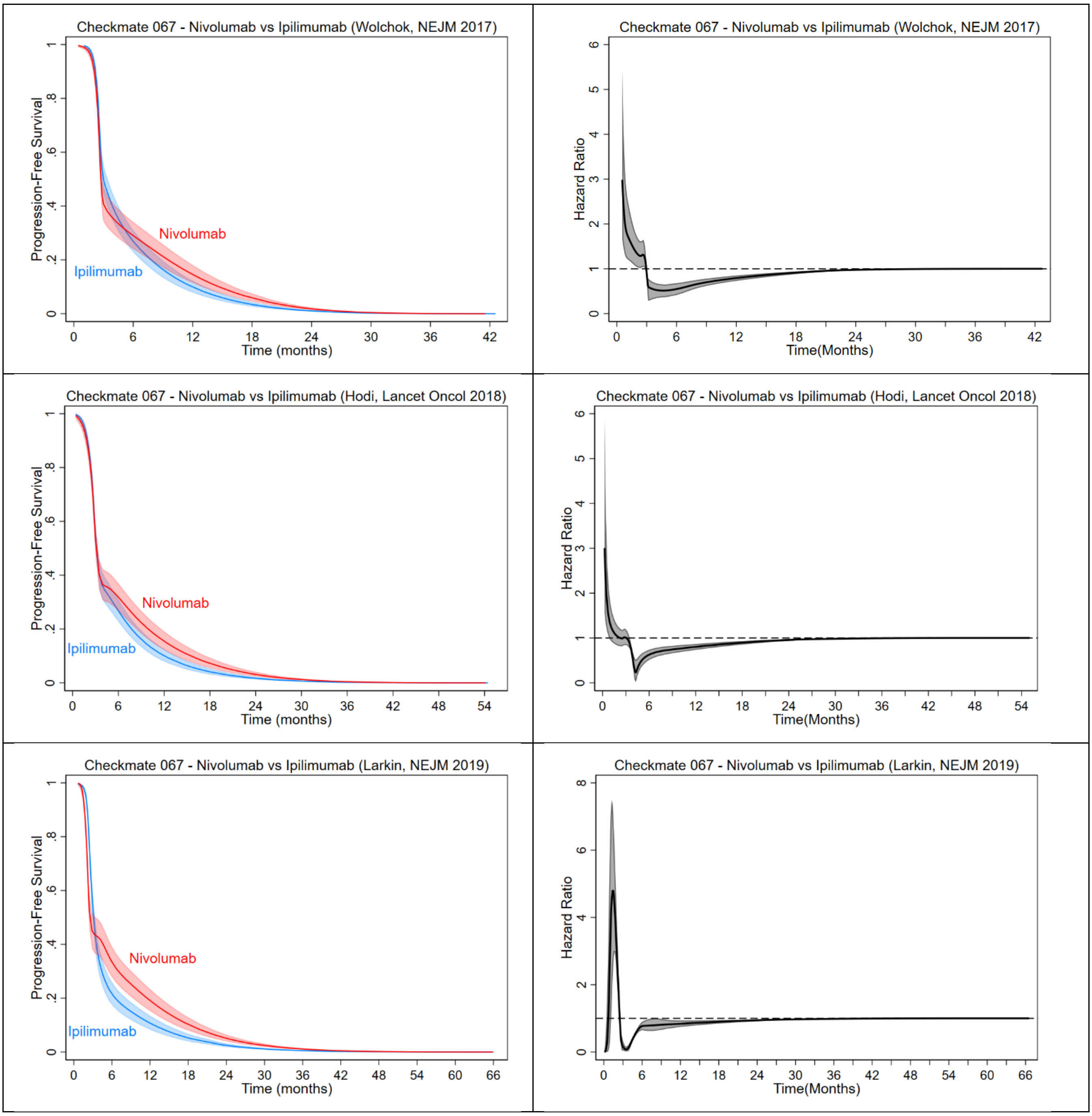

eFigure 6 (continued): Non-long-term responder population for patients with melanoma A. Progression free survival B. Hazard ratio and 95% confidence intervals and FPCM curves (main model) for patients with melanoma

© 2021 Filleron T et al. *JAMA Network Open.*

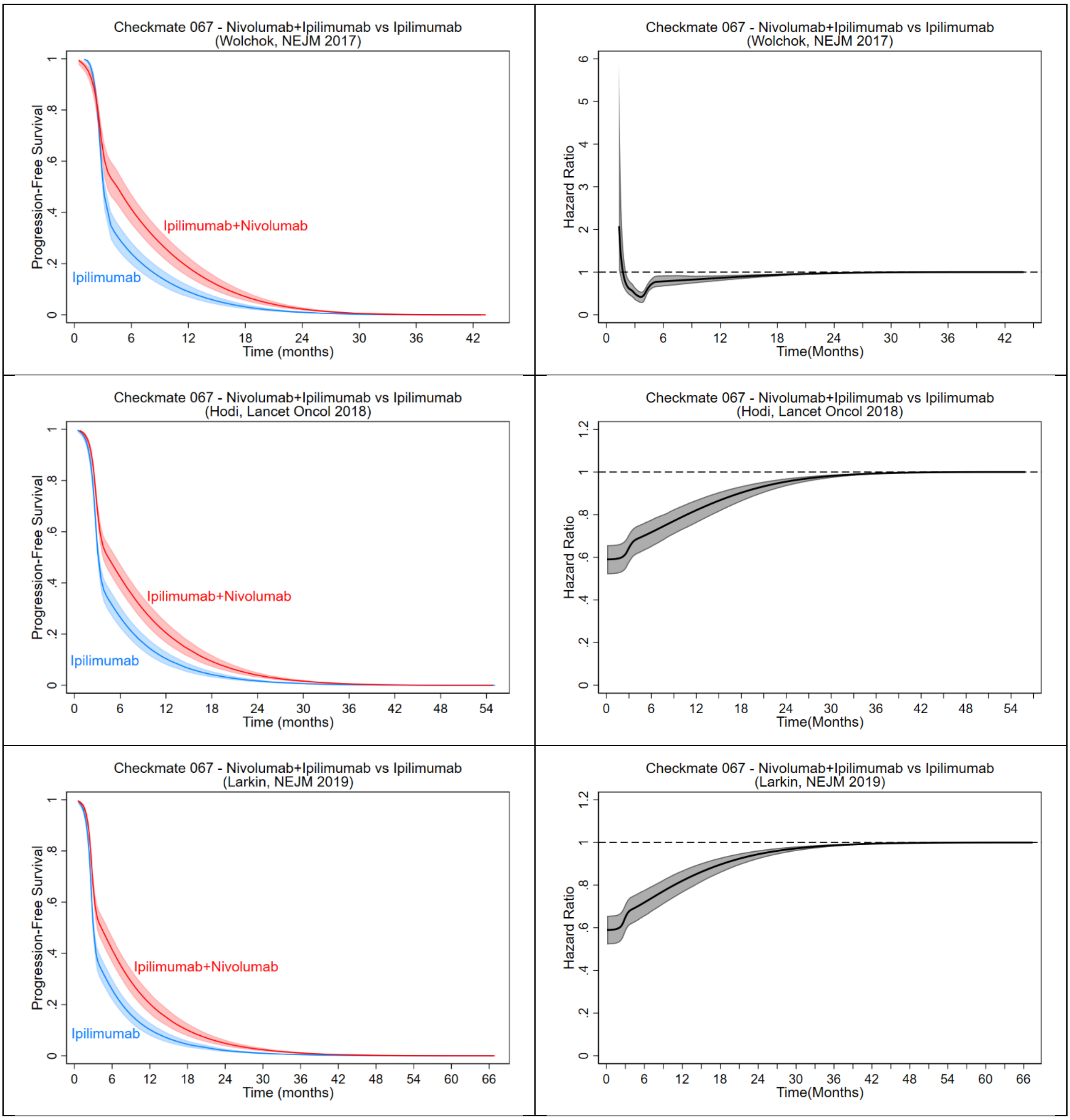

eFigure 6 (continued): Non-long-term responder population for patients with melanoma A. Progression free survival B. Hazard ratio and 95% confidence intervals and FPCM curves (main model) for patients with melanoma

© 2021 Filleron T et al. *JAMA Network Open.*

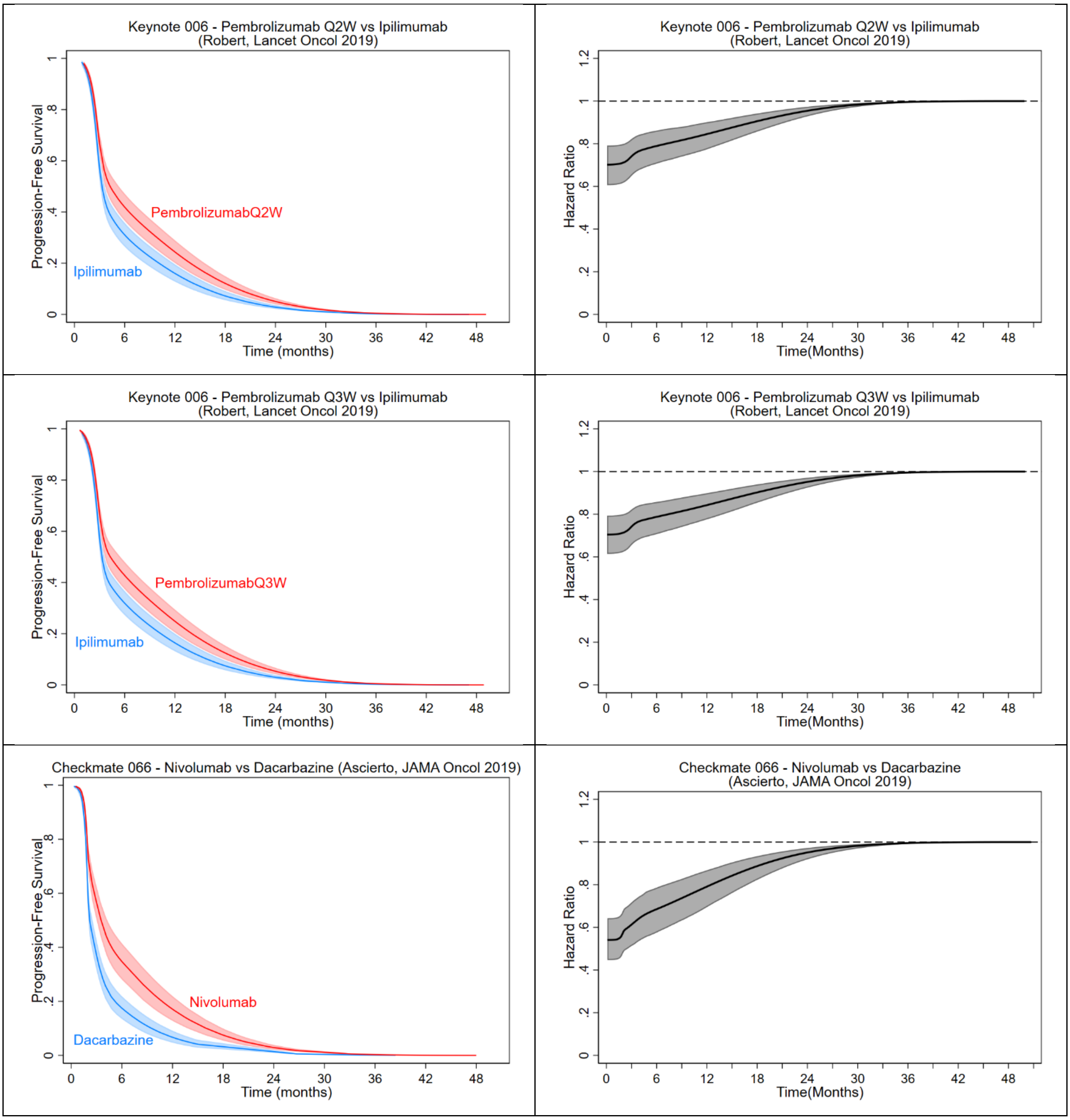

eFigure 6 (continued): Non-long-term responder population for patients with melanoma A. Progression free survival B. Hazard ratio and 95% confidence intervals and FPCM curves (main model) for patients with melanoma

© 2021 Filleron T et al. *JAMA Network Open.*

Non Small Cell Lung Cancer

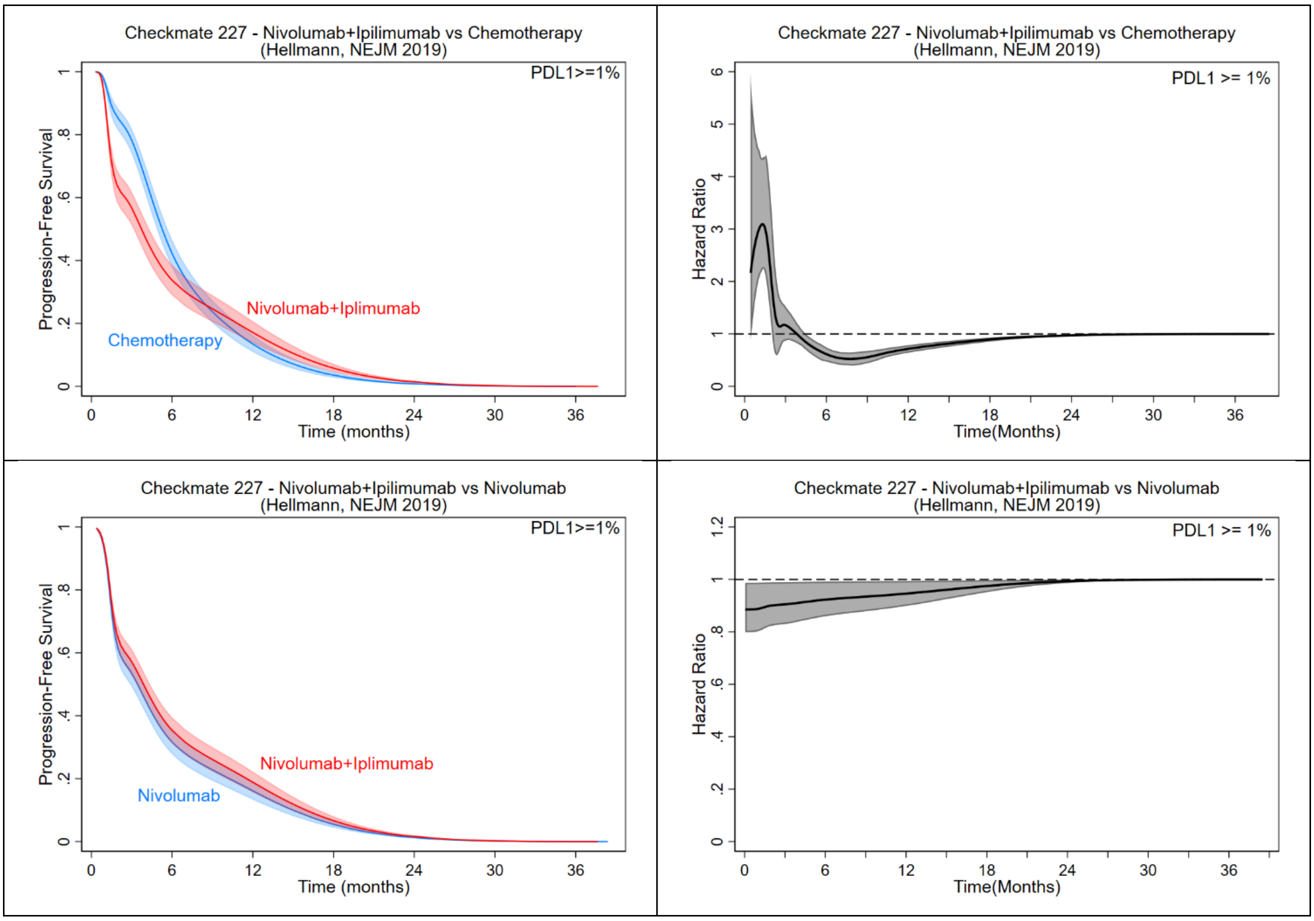

eFigure 7. Non-Long-Term Responder Population for patients with NSCLC

A. Progression Free Survival B. Hazard Ratio And 95% Confidence Intervals And FPCM Curves (Main Model) For Patients With Non-Small-Cell Lung Cancer

© 2021 Filleron T et al. *JAMA Network Open.*

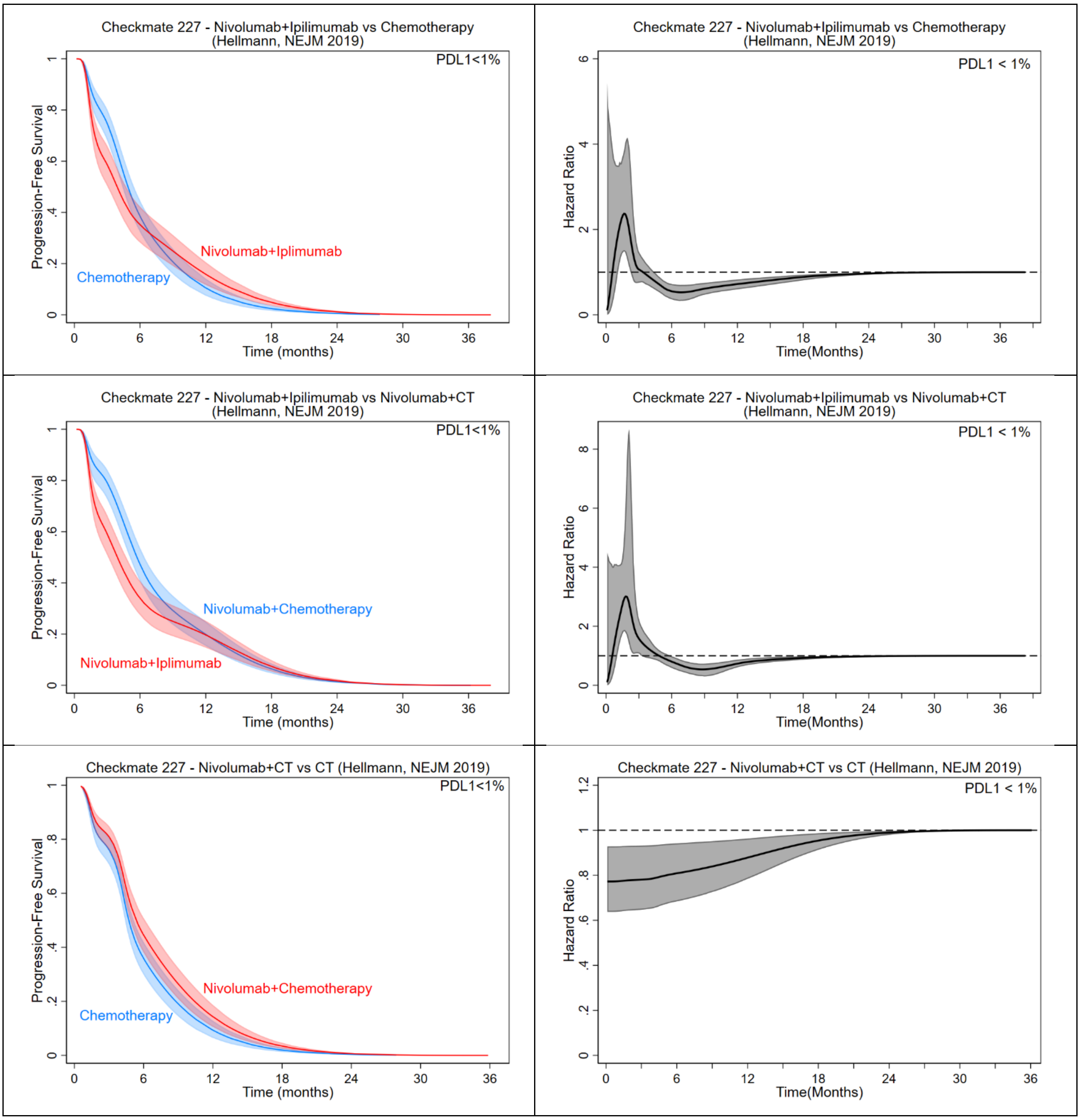

eFigure 7 (continued): Non-long-term responder population for patients with NSCLC A. Progression free survival B. Hazard ratio and 95% confidence intervals and FPCM curves (main model) for patients with non-small-cell lung cancer

© 2021 Filleron T et al. *JAMA Network Open.*

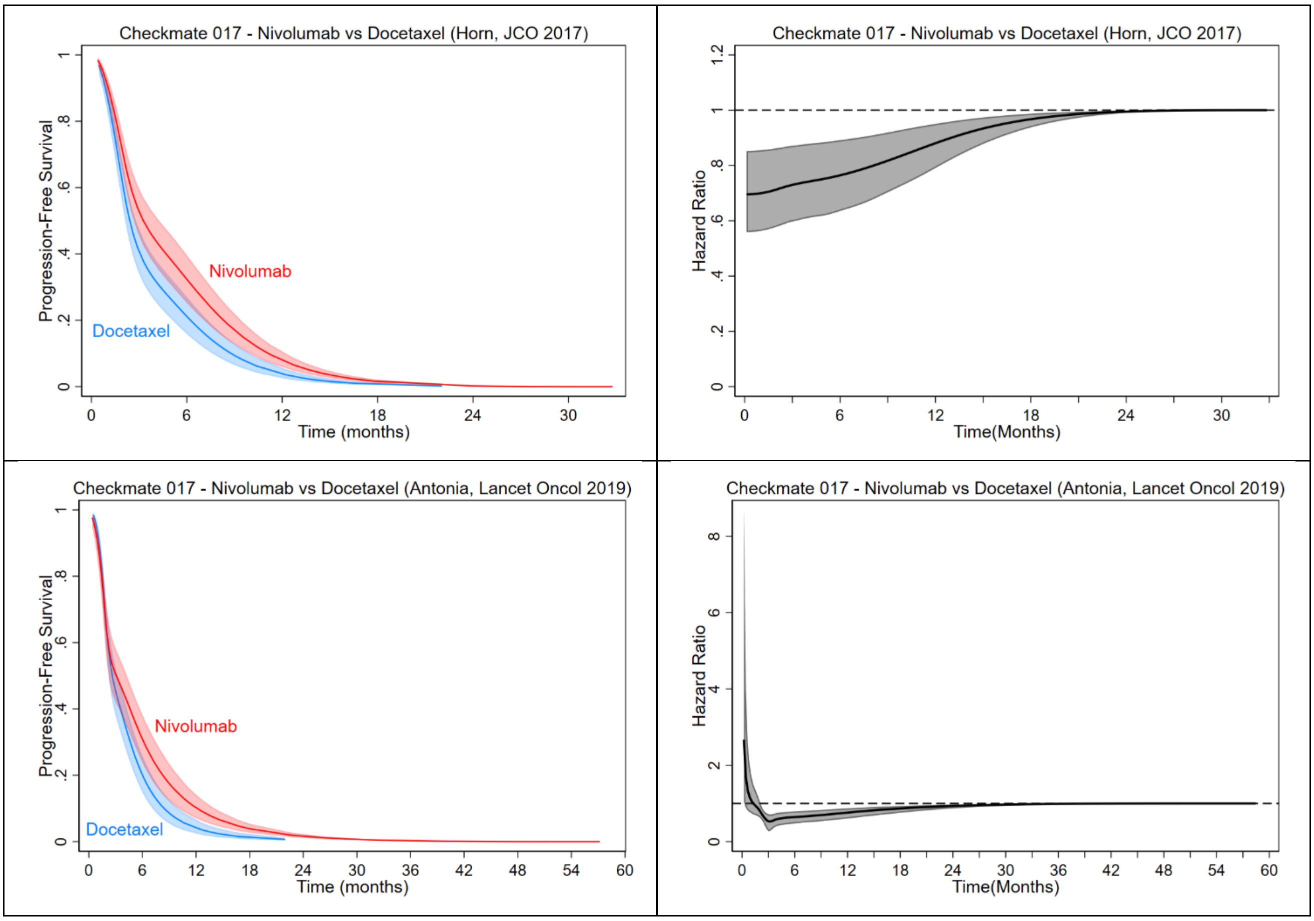

eFigure 7 (continued): Non-long-term responder population for patients with NSCLC A. Progression free survival B. Hazard ratio and 95% confidence intervals and FPCM curves (main model) for patients with non-small-cell lung cancer

© 2021 Filleron T et al. *JAMA Network Open.*

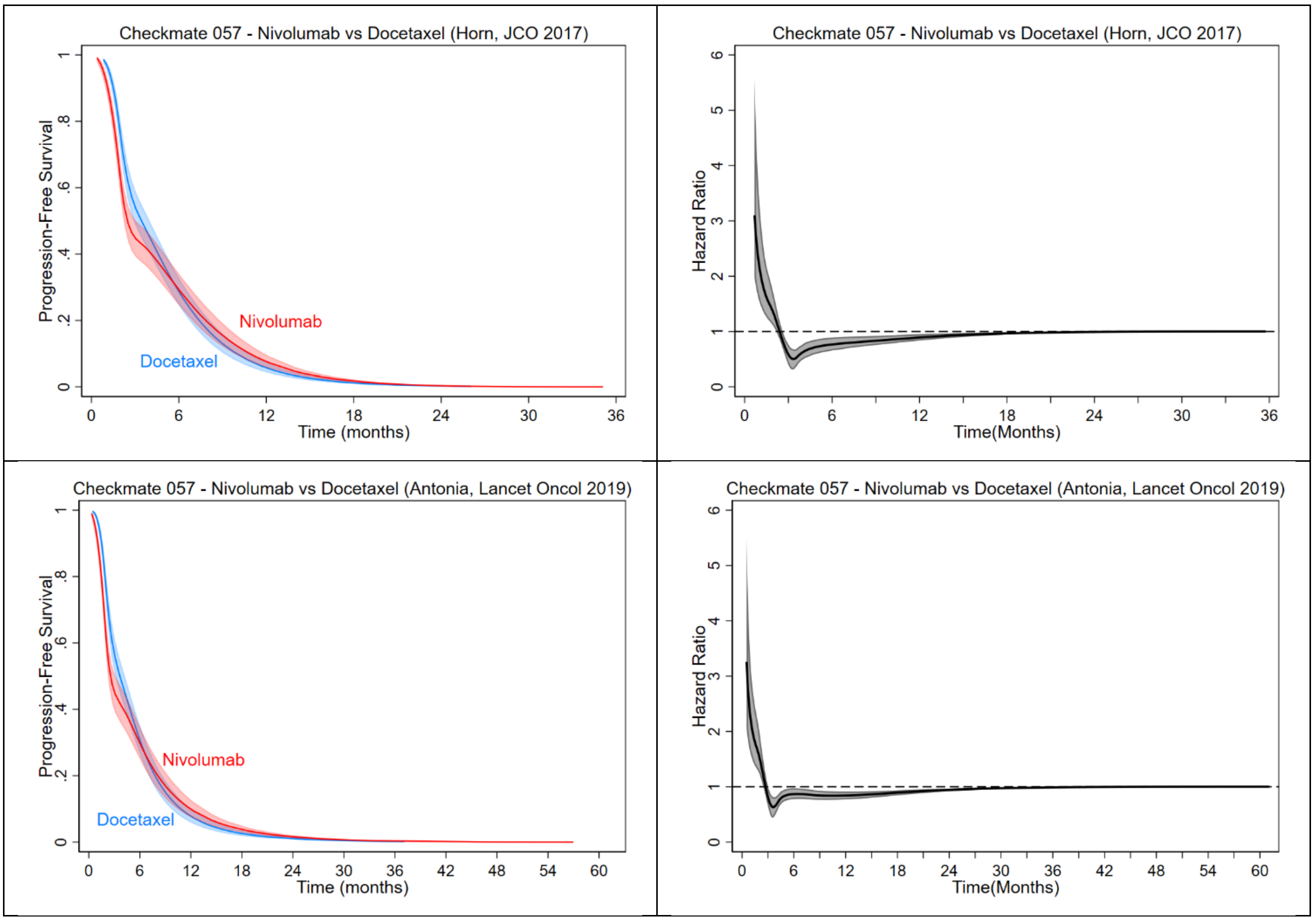

eFigure 7 (continued): Non-long-term responder population for patients with NSCLC A. Progression free survival B. Hazard ratio and 95% confidence intervals and FPCM curves (main model) for patients with non-small-cell lung cancer

© 2021 Filleron T et al. *JAMA Network Open.*

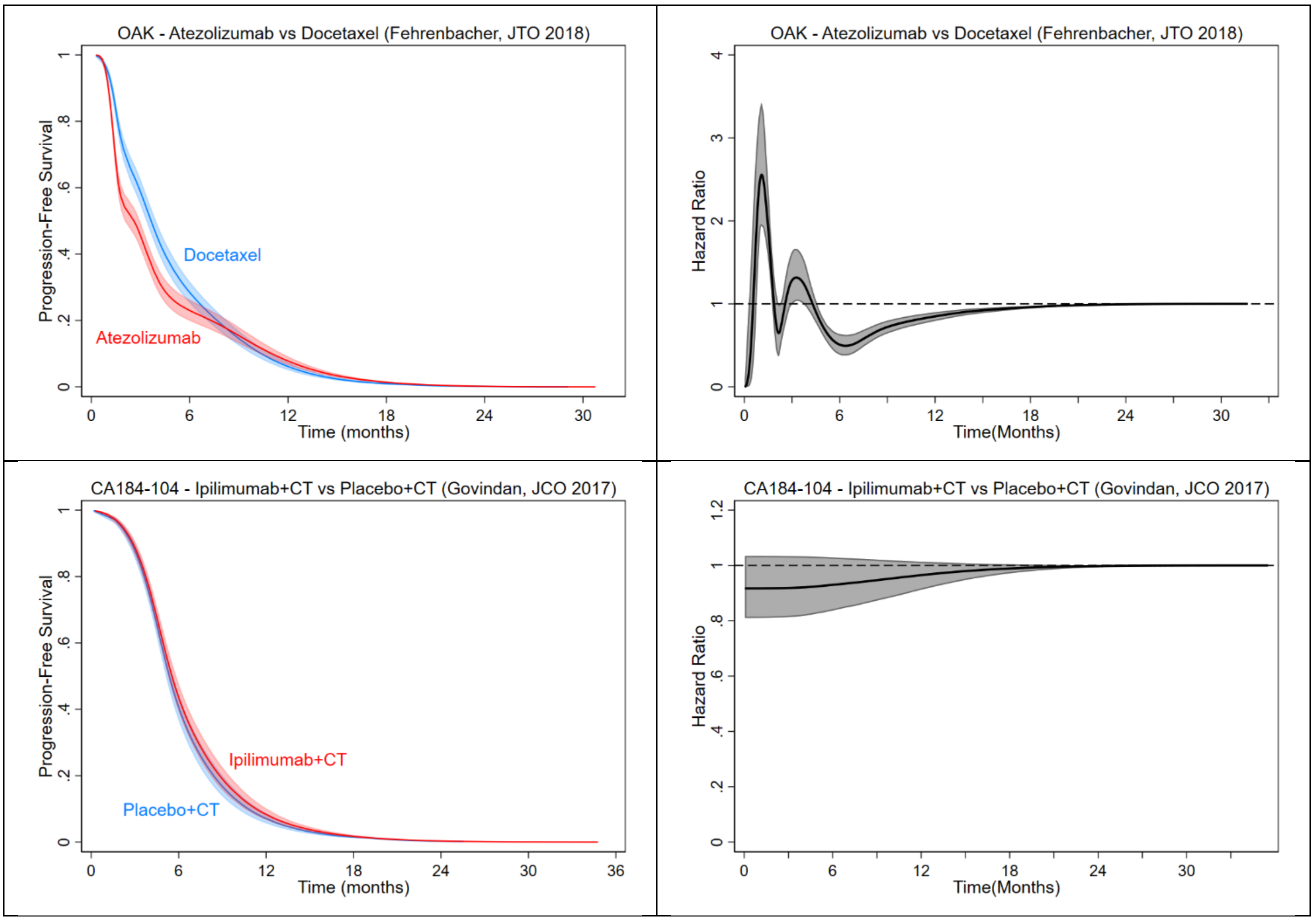

eFigure 7 (continued): Non-long-term responder population for patients with NSCLC A. Progression free survival B. Hazard ratio and 95% confidence intervals and FPCM curves (main model) for patients with non-small-cell lung cancer
